# Risk of Aneurysm Rupture (ROAR) study: protocol for a long-term, longitudinal, UK multicentre study of unruptured intracranial aneurysms

**DOI:** 10.1136/bmjopen-2022-070504

**Published:** 2023-03-16

**Authors:** Samuel Hall, Jacqueline Birks, Ian Anderson, Andrew Bacon, Paul M Brennan, David Bennett, Emmanuel Chavredakis, Giles Critchley, Graham Dow, Jonathan Downer, James Galea, Patrick Grover, Nihal Gurusinghe, Adel Helmy, Gueorgui Kounin, Nitin Mukerji, Hiren Patel, Jash Patel, Nicholas Ross, Jerome St George, Mario Teo, Christos Michael Tolias, Nikolaos Tzerakis, Christopher Uff, Janneke van Beijum, Kristin Veighey, Edward White, Peter Whitfield, Diederik Oliver Bulters, ROAR Investigators, Frederick Ewbank

**Affiliations:** 1 Department of Neurosurgery, University Hospital Southampton NHS Foundation Trust, Southampton, UK; 2 Centre for Statistics in Medicine, University of Oxford, Oxford, UK; 3 Department of Neurosurgery, The Leeds Teaching Hospitals NHS Trust, Leeds, UK; 4 Department of Neurosurgery, Sheffield Teaching Hospitals NHS Foundation Trust, Sheffield, UK; 5 Department of Clinical Neurosciences, Western General Hospital, Edinburgh, UK; 6 Department of Neurosurgery, NHS Tayside, Dundee, UK; 7 Department of Neurosurgery, Walton Centre NHS Foundation Trust, Liverpool, UK; 8 Department of Neurosurgery, University Hospitals Sussex NHS Foundation Trust, Brighton, UK; 9 Department of Neurosurgery, Nottingham University Hospitals NHS Trust, Nottingham, UK; 10 Neurosurgical Department, University Hospital of Wales Healthcare NHS Trust, Cardiff, UK; 11 National Hospital for Neurology and Neurosurgery, University College London Hospitals NHS Foundation Trust, London, UK; 12 Department of Neurosurgery, Lancashire Teaching Hospitals NHS Foundation Trust, Preston, UK; 13 Department of neurosurgery, Cambridge University Hospitals NHS Foundation Trust, Cambridge, UK; 14 Department of Neurosurgery, Hull University Teaching Hospitals NHS Trust, Hull, UK; 15 Department of Neurosurgery, South Tees Hospitals NHS Foundation Trust, Middlesbrough, UK; 16 Department of Neurosurgery, Northern Care Alliance NHS Foundation Trust, Salford, UK; 17 Department of Neurosurgery, Oxford University Hospitals NHS Foundation Trust, Oxford, UK; 18 Department of Neurosurgery, Newcastle Hospitals NHS Foundation Trust, Newcastle, UK; 19 Department of Neurosurgery, Institute of Neurological Sciences, NHS Greater Glasgow and Clyde, Glasgow, UK; 20 Department of Neurosurgery, North Bristol NHS Trust, Bristol, UK; 21 Neurosurgery, King's College Hospital NHS Foundation Trust, London, UK; 22 Department of Neurosurgery, University Hospitals of North Midlands NHS Trust, Stoke-on-Trent, UK; 23 Department of Neurosurgery, Barts Health NHS Trust, London, UK; 24 Department of Renal Medicine, University Hospital Southampton NHS Foundation Trust, Southampton, UK; 25 Department of Neurosurgery, University Hospitals Birmingham NHS Foundation Trust, Birmingham, UK; 26 South West Neurosurgery Centre, University Hospitals Plymouth NHS Trust, Plymouth, UK; 27 University Hospital Southampton NHS Foundation Trust, Southampton, UK

**Keywords:** epidemiology, stroke, neurosurgery

## Abstract

**Abstract:**

**Introduction:**

Unruptured intracranial aneurysms (UIA) are common in the adult population, but only a relatively small proportion will rupture. It is therefore essential to have accurate estimates of rupture risk to target treatment towards those who stand to benefit and avoid exposing patients to the risks of unnecessary treatment. The best available UIA natural history data are the PHASES study. However, this has never been validated and given the known heterogeneity in the populations, methods and biases of the constituent studies, there is a need to do so. There are also many potential predictors not considered in PHASES that require evaluation, and the estimated rupture risk is largely based on short-term follow-up (mostly 1 year). The aims of this study are to: (1) test the accuracy of PHASES in a UK population, (2) evaluate additional predictors of rupture and (3) assess long-term UIA rupture rates.

**Methods and analysis:**

The Risk of Aneurysm Rupture study is a longitudinal multicentre study that will identify patients with known UIA seen in neurosurgery units. Patients will have baseline demographics and aneurysm characteristics collected by their neurosurgery unit and then a single aggregated national cohort will be linked to databases of hospital admissions and deaths to identify all patients who may have subsequently suffered a subarachnoid haemorrhage. All matched admissions and deaths will be checked against medical records to confirm the diagnosis of aneurysmal subarachnoid haemorrhage. The target sample size is 20 000 patients. The primary outcome will be aneurysm rupture resulting in hospital admission or death. Cox regression models will be built to test each of the study’s aims.

**Ethics and dissemination:**

Ethical approval has been given by South Central Hampshire A Research Ethics Committee (21SC0064) and Confidentiality Advisory Group support (21CAG0033) provided under Section 251 of the NHS Act 2006. The results will be disseminated in peer-reviewed journals.

**Trial registration number:**

ISRCTN17658526.

**Strengths and limitations of this study**

- The unruptured intracranial aneurysm (UIA) treatment rate is lower in the UK than in many other high-income countries, which reduces selection bias and allows observation of the true UIA natural history.
- The large cohort size will allow inclusion of more covariates in prediction models than has been possible in previous studies including rare, but salient, patient groups such as those with autosomal dominant polycystic kidney disease.
- The UK has uniform medical coverage and given virtually all patients who suffer subarachnoid haemorrhage seek medical help, the strategy to use national databases for hospital admissions and deaths in a defined population provides a robust method for identification of outcome events with minimal loss to follow-up.
- This design makes the Risk of Aneurysm Rupture study an order of magnitude larger than previous natural history studies and allows for repeated follow-up and generation of true long-term rupture risk.
- Identifying rupture events from hospital admissions databases is reliant on diagnosis coding accuracy; this limitation has been mitigated by searching a broad range of possible codes for intracranial haemorrhagic events and subsequent diagnosis verification against original imaging studies.

## Introduction

 Unruptured intracranial aneurysms (UIA) are common in the general population with an estimated prevalence of 2.3%–3.2%.^1 2^ Aneurysm rupture resulting in subarachnoid haemorrhage is much less common with an annual incidence of 9 per 100 000 of the population.^3^ It is estimated that 1.4% of UIA rupture per year.^4^ Subarachnoid haemorrhage is a serious complication of UIA with a mortality rate of up to 67% and half of the survivors are left disabled.^5^ UIA can be prophylactically treated to prevent rupture, however these procedures carry at least a 5% risk of complications.^6^ In the absence of randomised controlled trial (RCT) data, the decision on proceeding to prophylactic treatment is dependent on natural history data. Decisions regarding whether to follow-up untreated patients radiologically also depend on our understanding of these data.

The first natural history study, and the most applicable to the UK population, was the International Study of Unruptured Intracranial Aneurysms (ISUIA).^7^ However, concerns over the data are well documented,^8^ and the generalisability of the results may be undermined by selection bias resulting from a high treatment rate of 71%. Five further natural history studies have been conducted,^9^,^13^ all in different populations with different selection biases and different periods of follow-up, and yielding different results. For example, Juvela *et al*^9^ reported rupture rates of 26% of UIA <7 mm over 30 years compared with 0% in similar aneurysms extrapolated from ISUIA. This difference may reflect a higher risk in the Finnish population or difference in study methodology—it is not known which.

These six studies were combined in an individual patient-level meta-analysis as the PHASES score, which provides an estimate for 5-year rupture risk.^4^ The PHASES score is the best available evidence for UIA rupture risk. However, it has never been externally validated, and particularly given the heterogeneity in the underlying studies, there is an urgent need to do so.

Furthermore, PHASES was limited to the risk factors available for analysis from the underlying studies. There are many more patient and aneurysm features which have been shown to be associated with rupture or that may be hypothesised to predispose to rupture. These range from common modifiable variables like smoking, to rarer non-modifiable ones like family history and autosomal dominant polycystic kidney disease (ADPKD).

One of the main shortfalls of PHASES is that, with one exception, the constituent studies are based on short lengths of follow-up, with the majority of patients followed up just 1 year, which has been used to generate 5-year risks in PHASES. Clinicians further extrapolate PHASES to patient’s lifetime risk which all makes the large assumption that risk does not change over time. Moreover, even if the bleeding risk remains constant over time, any seemingly small inaccuracies in short-term estimates can become very significant when extrapolated over many decades.

We therefore designed a large multicentre longitudinal study of patients with UIAs to address these concerns.

## Methods and analysis

### Objectives

This study has three objectives:

1. To measure the accuracy of the PHASES score at predicting UIA rupture rates in the UK population.
2. To develop a new, more personalised, predictive model for aneurysm rupture incorporating additional covariates thought to influence risk.
3. To measure aneurysm rupture risk over time periods >5 years.

### Study setting

This is a multicentre study conducted at up to 30 tertiary neurosurgery units in the UK. Patients will be identified by the neurosurgery unit who diagnosed their UIA. Each unit will collect their baseline data on patients’ clinical and aneurysm characteristics from the time of diagnosis. Central searches of hospital admissions databases, and data analysis, will be performed by the coordinating team at University Hospital Southampton NHS Foundation Trust and the University of Oxford. A separate cohort enriched in patients with autosomal dominant polycystic kidney disease will be established using similar methodology, from up to 70 renal units in the UK.

### Study design

The Risk of Aneurysm Rupture (ROAR) study is a longitudinal study that uses a hybrid design of patient identification at regional neurosurgical units and prospectively collected national hospital admissions databases for outcome events. The study will establish a cohort of patients with a UIA and measure how many subsequently ruptured. This observed rupture rate can be compared with a rate estimated by the PHASES score to determine its accuracy.

Each neurosurgery unit in the UK will be invited to search their medical records for patients diagnosed with a UIA. This search method will be tailored by the individual neurosurgery unit based on what records they keep, but search strategies may include: multidisciplinary team (MDT) meeting logs, radiology reports or electronic patient records. The search strategy will be predefined by individual units dependent on their record systems. The maximum date range for identifying patients is documents dated 1 January 2006 to 31 December 2020, however, this period may be shorter for each unit depending on availability of records. Whatever date range is chosen by the unit, it will be predefined and if the patient’s UIA was newly diagnosed during this period it will be classed as *new* and those who were diagnosed before this period but identified from a document during the aneurysm follow-up will be classed as *follow-up*, and their recruitment date recorded as the date of the document from which they were identified. This will minimise the prevalence-incidence (Neyman)^14^ selection bias created by identifying patients diagnosed before the unit’s search period but who survive without rupture to make it into the search period, whereas their counterparts diagnosed at the same time who rupture and die are not identified. It will also allow comparison of rupture risk of newly diagnosed aneurysms and those with known diagnoses. Baseline clinical characteristics and aneurysm characteristics will be collected as per the common data elements for UIA research.^15^ All data collectors will undergo training in coding data elements delivered by the coordinating centre. Collecting baseline data from local medical records allows deeper phenotypic typing and higher data fidelity than using national admission databases.

Patient identifiable details (name, date of birth, post code, NHS/CHI number) will be securely sent by each neurosurgery unit to the coordinating centre for consolidation and linkage to the national databases for hospital admissions. These databases are: Hospital Episode Statistics (HES), Patient Episode Database for Wales (PEDW) and Scottish Morbidity Database (SMD). These databases also link to the Civil Registrations—Death and National Registry Scotland for death records. Patients will be linked to hospital admissions based on International Classification of Diseases 10th Revision (ICD-10) diagnosis codes for intracranial haemorrhages and OPCS4 codes for aneurysm occlusion treatments. These databases record every hospital admission in their respective country and thus using this as the outcome source, combined with death records, will allow identification of every aneurysmal subarachnoid haemorrhage (aSAH) regardless of whether they were managed in a neurosurgery unit, a district general hospital, migrated out of the region in which their UIA was diagnosed, or died in the community. The number of patients with aSAH who are not captured by this method, either because they do not present to hospital or emigrate out of the UK, is expected to be very small. Rupture rates can be adjusted based on national emigration rates.

Patients will be censored if there is any occlusive treatment of the UIA or patient death. Occlusive treatment includes either microsurgical or endovascular techniques either partial or complete. If none of these censoring events are observed, then they will be censored on the day the cohort is submitted to the HES/PEDW/SMD databases.

The ICD-10 codes to be searched for rupture events have been selected in accordance with the UK Biobank stroke research and include: aSAH (I60.1-9), intracerebral haemorrhage (I61.0-9), traumatic subarachnoid haemorrhage (S06.6) and spontaneous subdural haemorrhage (I62.00-I62.02).^16^ The use of codes beyond those for aSAH will capture any hospital admissions for aneurysm rupture which have resulted in other forms of intracranial bleed or have been miscoded.

It is expected that the use of codes beyond that for just aSAH will return many admissions not due to aneurysm rupture. The matched hospital admissions, and death records, will be returned to the coordinating unit who will in turn use pseudonymisation numbers to inform the respective local unit of their patient’s admission for possible aSAH. The local unit will review the imaging studies, discharge summaries and death certificates for these admissions and confirm or refute the diagnosis. Statistical analysis will begin once the diagnosis for all of the matched admissions is confirmed.

The study is currently opening new sites and identifying patients for inclusion. The baseline data collection is planned to finish by 31 July 2023 and the results released no sooner than 31 July 2024. The study end date is currently 31 July 2034 to allow for repeated searching of hospital admissions databases in the future thereby further extending the follow-up period.

### Eligibility criteria

#### Inclusion

1. Age 18 years or older.
2. Intracranial, intradural, unruptured aneurysm.
3. Aneurysm confirmed on cranial angiogram - Computerised Tomography Angiogram (CTA)/ Magnetic Resonance Angiography (MRA)/ Digital Subtraction Angiography (DSA).
4. Identification of UIA from records between 1 January 2006 and 31 December 2020.

#### Exclusion

1. Mycotic or vasculitic aneurysms.
2. Aneurysm diagnosed on CT or MRI alone.
3. Arterio-Venous Malformation (AVM) associated flow aneurysms.
4. Extradural aneurysms (eg, intracavernous).
5. Aneurysms treated by either microsurgical or endovascular techniques before the search period.
6. Small lesions uncertain as to whether they are truly aneurysmal (‘dilatation’, ‘bulge’, ‘infundibulum’).

### Outcomes

#### Primary end points

The primary end point is rupture of an untreated UIA at a timepoint at least 1 day following diagnosis. A rupture event is defined as either radiological evidence of aSAH in a distribution consistent with the aneurysm location, CSF spectrophotometry positive for xanthochromia per the local unit’s reference range or death certificate stating subarachnoid haemorrhage in causes of death 1a-c.

#### Secondary end points

The secondary end point is aneurysm growth on follow-up imaging. Recruiting units will record if patients have undergone follow-up imaging. Aneurysm growth will be recorded if there was any clinically observable growth, in the opinion of a consultant neuroradiologist or an MDT, when directly comparing baseline and follow-up scans.

### Data transmission and editing

The recruiting units will populate two data sheets, one containing patient identifiable details required for hospital admission database searches and a second containing clinical details only. These two spreadsheets will be cross-referenced using an aneurysm-level pseudonymisation number contained in both spreadsheets. Recruiting units will send each data sheet to the coordinating unit separately through a 256-bit end-to-end encryption service.

The requirement for editing the data will be minimised through the use of restricted fields and predefined lists of valid codes for each element on the datasheet. All queries and discrepancies raised by the coordinating centre regarding the data entry will be submitted to the respective recruiting units through a single query sheet referencing the pseudonymisation number.

### Sample size

There are no accepted methods for power calculation for validation studies of prognostic models. Earlier methods included the rule of thumb to have 10 events for every covariate tested, however, more modern methods for minimum sample size calculation have been proposed by Riley *et al.*^17^ The online package *pmsampsize* uses the method by Riley *et al* to estimate the minimum sample size. Using figures from our feasibility work (2124 patients with 60 rupture events over 4010 patient years), estimating 28 df and varying Cox-Snell R^2^ value d from 0.03 to 0.05 resulted in a minimum sample size ranging from 5143 to 8559. The number of df allows for all categorical variables as well as continuous variables such as patient age or aneurysm size which may require polynomial equations.

The method by Riley *et al* is yet to be widely used and does not consider the prevalence of uncommon variables such as ADPKD. The older rule of thumb requiring 10 events per covariate was therefore also considered. For the first objective, the 6 covariates in the PHASES score will be tested suggesting at least 60 SAHs will need to be captured. ISUIA recorded 51 ruptures in 1692 patients over 4.1 years. Sixty events may therefore be expected in 1990 patients with 8161 years of follow-up. For the second objective, 120 events will need to be observed to account for the six additional commonly occurring covariates which would be expected in 3981 patients with 16 332 years of follow-up.

However, the 10 events per covariate rule of thumb does not consider the prevalence of the covariate in the study population. Therefore, rarely occurring populations may have insufficient data to estimate risk. ADPKD is one such population and including it as a covariate requires a larger study size. In ADPKD, 10 events are expected in 1360 patient years of follow-up assuming the risk of SAH is similar to the general population. However, 16 332 years follow-up would only yield 195 years in patients with ADPKD (based on a population study which found 53/4436 patients with UIA had ADPKD).^18^ Therefore, 113 905 years of follow-up would be required to capture 10 ruptures among 1340 years follow-up in patients with ADPKD. This equates to a total cohort of 22 781 patients.

The Transparent Reporting of a multivariable Prediction model for individual Prognosis or Diagnosis (TRIPOD) guidelines highlight the lack of consensus of how to calculate a sample size and suggest aiming for larger sample sizes which give more precise and reliable results. Smaller sample sizes are at risk of performance optimism. Therefore, the ROAR study will aim to collect 20 000 patients. Based on feasibility studies, this is the maximum practical sample size, and power calculations show that it is sufficient to generate precise estimates and account for all covariates.

### Statistical analysis

#### Objective 1: PHASES validation

The PHASES study provides the coefficients from their Cox regression model and baseline survival at 5 years which allows the absolute 5-year risk of rupture to be calculated for all patients who are not censored before 5 years. Time to censoring will be calculated (whichever is soonest of the date of treatment, date of death or the HES/PEDW/SMD search date) to ensure 5 years of follow-up if rupture has not occurred. Discrimination will be assessed using Harrell’s C-index of concordance and Royston and Sauerbrei’s D statistic. Calibration will be assessed at the 5-year time point using the method by Royston.^19^ These will be used to calculate the number of SAH events per 5 years for each PHASES score (≤2 to 12+) and expressed as a percentage with 95% CI to compare with the PHASES estimates.

#### Objective 2: additional prognostic factors

A new risk prediction model for rupture will be developed using the total data set, including the additional possible risk factors. The Cox regression model will be used for risk of rupture. The absolute risks can be estimated at relevant time intervals, 2, 5 and 10 years. Numbers of missing values will be summarised for each factor. Multiple imputation will be used to replace missing values. Discrimination of the final model will be assessed with Harrell’s C-statistic. Internal validity will be assessed by bootstrap resampling.

#### Objective 3: long-term rupture rates

All patients, including those who underwent aneurysm occlusion, will be included in time-to-event analysis which will cover the whole duration of available follow-up. This will include Kaplan-Meier and proportional hazards models for univariate and multivariate survival curve fitting. A cumulative hazard plot will be used to assess if rupture risk is constant or varies with time from diagnosis.

Once the cohort is established, funding will be sought for repeated searches at regular intervals (5 yearly) to update models and provide progressively long-term rupture rates.

### Patient and public involvement

A workgroup was organised with the Wessex Subarachnoid Haemorrhage Support Group to discuss UIA research where it was confirmed that better decision making on aneurysm treatment is the main concern for patients, but patients do not want to have their management randomised and therefore an RCT is unlikely to succeed. Consequently, a better understanding of the natural history of UIA was deemed the top priority and that long-term, ideally lifetime risks, are what is relevant to patients. This formed the basis of the current study.

During May 2020, while face-to-face public involvement was not possible due to the COVID-19 pandemic, patients in the neurovascular telephone clinic at University Hospital Southampton were surveyed to assess the study design. All patients strongly supported a study of the natural history of UIA. Although some said they would decline participation in imaging or interventional studies, all confirmed they would be happy for their records to be searched for a natural history study, without full informed consent as is proposed.

## Ethics and dissemination

### Ethical considerations (including informed consent)

Seeking informed consent from all patients to break confidentiality and transfer their identifiable details is not possible without biasing the results. Patients whose aneurysm ruptures have a high likelihood of death or severe disability which would leave them unable to provide informed consent. If informed consent was mandatory, the final cohort would contain an under-representation of patients whose aneurysm ruptured, thus skewing the observed rupture rates.

In order to process patient identifiable data without consent, the study has been given conditional support under Section 251 from the HRA Confidentiality Advisory Group (21/CAG/0033). This allows the transfer of patient identifiable data outside of the direct clinical care team for the purpose of this study. The patient identifiable data can thus be transferred to the coordinating team who in turn can upload these data to the HES/PEDW/SMD databases. The protocol has also been reviewed by the South Central Hampshire A Research Ethics Committee (REC) and issued a favourable opinion in March 2021 (21/SC/0064). The REC and CAG committees will be updated on all significant protocol amendments by the study coordinator.

### Monitoring

As a study without direct patient contact there will not be a separate data monitoring committee. Instead, this role will be conducted by the trial management committee.

### Dissemination and data availability

The results will be disseminated in peer-reviewed journals. Authorship will follow International Committee of Medical Journal Editors recommendations and professional writers will not be used. On completion of the study, the anonymised dataset will be available both to members of the ROAR collaboration and other external researchers. They will be available from the chief investigator on reasonable request.

## Discussion

Although the natural history of UIA has been previously investigated with multiple prospective cohort studies, the rupture rate has varied significantly between these. The design of the ROAR study addresses many of the criticisms of these previous natural history studies. ISUIA^7^ is the first natural history study and is drawn from a population that is genetically closest to that of the UK. However, its results are subject to selection bias with 71% of their UIA being treated either before inclusion or during follow-up. It is not known if that selection was random or based on a feature associated with risk such as aneurysm irregularity. It is therefore not known how the rupture rates in the remaining potentially lower-risk patients translate to the general population. The UK has a much lower treatment rate of UIA (approximately 20%). This is one of the lowest rates in a high-income country making it the ideal setting for a natural history study. It remains however that there will be some treatments performed during the study which inevitably will produce some selection bias that cannot be eliminated. Other countries with lower treatment rates are likely to also have low availability of imaging and hence much lower case identification limiting the cohort size and introducing different biases.

There is also a risk of selection bias in ROAR arising from the methods employed for patient identification. Patients in whom no MDT, clinic notes or radiology report was created would be effectively excluded. These are also the patients more likely to not undergo treatment. However, our survey of UK neurovascular surgeons suggest it is only a very small minority that are not discussed at MDT or seen in a neurosciences clinic. This was also borne out in pilot studies in the development of the protocol. The timeframe for searching patients within a unit’s available records creates the potential for introducing prevalence-incidence selection bias. The ROAR study will mitigate this by identifying patients as *new* or *follow-up* based on whether or not the UIA was diagnosed during that unit’s search window and use the identifying document dates accordingly.

The largest UIA studies are based on Japanese populations and the study with the least bias on a Finnish population. Both observed higher rates of aneurysm rupture than ISUIA. Although it has been assumed ethnicity is a risk factor for rupture and therefore included in the PHASES score, it is not known if the different rupture rates in these studies were due to differences in genetics, environment or study design (and consequently biases). The ROAR study will observe rupture rates in a UK population which removes any concerns over the influence of ethnicity in the results and makes it generalisable for UIA decision making in patients in the UK. Given the use of national-level databases, ROAR is designed as a single country study and as a result there are limitations to its generalisability outside the UK. However, the UK’s population probably more closely resembles most European and North American countries than Japan or Finland.

With the exception of the study by Juvela *et al*,^9^ the follow-up lengths of previous UIA natural history studies are <5 years. Patients are generally not interested in such short-term risks and want to know their lifetime risk. Unfortunately, it is not known if risk is constant over time and therefore if these short-term estimates can be extrapolated to a patient’s lifetime. The hybrid design of the ROAR study will immediately generate follow-up periods of up to 15 years per patient. These will still be shorter than the typical 30-year life expectancy of someone diagnosed with a UIA at 50 and there will still therefore be limitations to extrapolation. However, it will be possible to perform repeat searches of the prospectively maintained databases for hospital admissions and deaths at intervals in the future such that ultimately it will yield realistic lifetime estimates.

Long-term follow-up of such a large cohort of patients is only feasible through the use of national databases. A traditional prospective study would be too costly and time consuming to be realistic as well as suffering significant loss to follow-up. The risk posed by using national databases is that patients emigrating are not censored when they leave the country. However, these databases record when patients deregister their general practice at which time they will be censored from further analysis.

One of the limitations of using national databases for hospital admissions is that identifying rupture events relies on the accuracy of the hospital coders and the diagnosis codes they assign (both to the primary and secondary diagnoses). The ROAR study will assess the magnitude of any miscoding by collecting any available follow-up data from the neurosciences record. In cases where a confirmed aneurysm rupture occurred, we will examine which hospital codes were assigned to that episode. We will also mitigate this limitation by searching for codes for all types of intra-cranial haemorrhage, not just subarachnoid haemorrhage, and subsequently review the medical records and imaging studies to confirm the true diagnosis for that patient.

While subject to a number of limitations, the ROAR study has mitigations for most of these and will therefore be less susceptible to them than previous studies. It is therefore expected to definitively evaluate the validity of PHASES, assess additional predictors of rupture and assess long term risks of rupture.

## Data Availability

Data are available on reasonable request.
