# Supplementary material for: Risk of Aneurysm Rupture (ROAR) study: protocol for a long-term, longitudinal, UK multicentre study of unruptured intracranial aneurysms
Source: BMJ Open. 2023 Mar 16;13(3):e070504. doi: 10.1136/bmjopen-2022-070504 (PMC10030903; doi:10.1136/bmjopen-2022-070504)
Supplement: Author's manuscript [file bmjopen-2022-070504.draft_revisions.pdf]

BMJ Open

BMJ Open is committed to open peer review. As part of this commitment we make the peer review history of every article we publish publicly available.

When an article is published we post the peer reviewers' comments and the authors' responses online. We also post the versions of the paper that were used during peer review. These are the versions that the peer review comments apply to.

The versions of the paper that follow are the versions that were submitted during the peer review process. They are not the versions of record or the final published versions. They should not be cited or distributed as the published version of this manuscript.

BMJ Open is an open access journal and the full, final, typeset and author-corrected version of record of the manuscript is available on our site with no access controls, subscription charges or pay-per-view fees (http://bmjopen.bmj.com).

If you have any questions on BMJ Open's open peer review process please email info.bmjopen@bmj.com

BMJ Open

A UK multicentre long term longitudinal study of unruptured intracranial aneurysms: the Risk Of Aneurysm Rupture (ROAR) Study

Journal:	*BMJ Open*
Manuscript ID	bmjopen-2022-070504
Article Type:	Protocol
Date Submitted by the Author:	24-Nov-2022
Complete List of Authors:	Hall, Samuel; University Hospital Southampton NHS Foundation Trust, Department of Neurosurgery<br>Birks, Jacqueline; University of Oxford, Centre for Statistics in Medicine<br>Anderson, Ian; The Leeds Teaching Hospitals NHS Trust, Department of Neurosurgery<br>Bacon, Andrew; Sheffield Teaching Hospitals NHS Foundation Trust, Department of Neurosurgery<br>Brennan, Paul; Western General Hospital, Department of Clinical Neurosciences<br>Bennett, David; NHS Tayside, Department of Neurosurgery<br>Chavredakis, Emmanuel; Walton Centre NHS Foundation Trust, Department of Neurosurgery<br>Critchley, Giles; University Hospitals Sussex NHS Foundation Trust, Department of Neurosurgery<br>Dow, Graham; Nottingham University Hospitals NHS Trust, Department of Neurosurgery<br>Downer, Jonathan; Western General Hospital, Department of Clinical Neurosciences<br>Galea, James; University Hospital of Wales Healthcare NHS Trust, Neurosurgical Department<br>Grover, Patrick; University College London Hospitals NHS Foundation Trust, National Hospital for Neurology and Neurosurgery<br>Gurusinghe, Nihal; Lancashire Teaching Hospitals NHS Foundation Trust, Department of Neurosurgery<br>Helmy, Adel; Cambridge University Hospitals NHS Foundation Trust, Department of neurosurgery<br>Kounin, Gueorgui; Hull University Teaching Hospitals NHS Trust, Department of Neurosurgery<br>Mukerji, Nitin; South Tees Hospitals NHS Foundation Trust, Department of Neurosurgery<br>Patel, Hiren; Northern Care Alliance NHS Foundation Trust, Department of Neurosurgery<br>Patel, Jash; Oxford University Hospitals NHS Foundation Trust, Department of Neurosurgery<br>Ross, Nicholas; Newcastle Hospitals NHS Foundation Trust, Department of Neurosurgery<br>St George, Jerome; NHS Greater Glasgow and Clyde, Institute of Neurological Sciences<br>Teo, Mario; North Bristol NHS Trust, Department of Neurosurgery

Tolias, Christos; King's College Hospital NHS Foundation Trust, Neurosurgery
Tzerakis, Nikolaos; University Hospitals of North Midlands NHS Trust, Department of Neurosurgery
Uff, Christopher; Barts Health NHS Trust, Department of Neurosurgery
van Beijum, Janneke; University Hospital of Wales Healthcare NHS Trust, Neurosurgical department
Veighey, Kristin; University Hospital Southampton NHS Foundation Trust, Department of Renal Medicine
White, Edward; University Hospitals Birmingham NHS Foundation Trust, Department of Neurosurgery
Whitfield, Peter; University Hospitals Plymouth NHS Trust, South West Neurosurgery Centre
Bulters, Diederik ; University Hospital Southampton NHS Foundation Trust, Department of Neurosurgery
ROAR Investigators, ROAR Investigators; University Hospital Southampton NHS Foundation Trust

Keywords: EPIDEMIOLOGY, Stroke < NEUROLOGY, NEUROSURGERY

SCHOLARONE™
Manuscripts

A UK multicentre long term longitudinal study of unruptured intracranial aneurysms: the Risk Of Aneurysm Rupture (ROAR) Study

Samuel Hall[1], Jacqueline Birks[2], Ian Anderson,[3] Andrew Bacon,[4] Paul Brennan,[5] David Bennett,[6] Emmanuel Chavredakis,[7] Giles Critchley,[8] Graham Dow,[9] Jonny Downer,[5] James Galea,[10] Patrick Grover,[11] Nihal Gurusinghe,[12] Adel Helmy,[13] Gueorgui Kounin,[14] Nitin Mukerji,[16] Hiren Patel,[17] Jash Patel,[18] Nicholas Ross,[19] Jerome St George,[20] Mario Teo,[21] Christos Tolias,[22] Nikolaos Tzerakis,[23] Christopher Uff,[24] Janneke van Beijum,[10] Kristin Veighey,[25] Edward White,[26] Peter Whitfield,[27] Diederik Bulters[1]

[1] Department of Neurosurgery, University Hospitals Southampton NHS Foundation Trust, Southampton, UK

[2] Centre for Statistics in Medicine, Medical Sciences Division, University of Oxford, Oxford, UK

[3] Department of Neurosurgery, The Leeds Teaching Hospitals NHS Trust, Leeds, LS1 3EX

[4] Sheffield Teaching Hospitals NHS Foundation Trust, Sheffield, S10 2JF

[5] Department of Clinical Neurosciences, NHS Lothian, Edinburgh, EH1 3EG

[6] Department of Neurosurgery, NHS Tayside, Dundee, DD2 1SG

[7] Department of Neurosurgery, Walton Centre NHS Foundation Trust, Liverpool, L9 7LJ

[8] Department of Neurosurgery, University Hospitals Sussex NHS Foundation Trust, Brighton, BN2 5BE

[9] Department of Neurosurgery, Nottingham University Hospitals NHS Trust, Nottingham, NG7 2UH

[10] Neurosurgical Department, Floor 4, Corridor B, University Hospital Wales, Heath Park, Cardiff CF14 4XW.

[11] National Hospital for Neurology and Neurosurgery, University College London Hospitals NHS Foundation Trust, London, WC1N 3BG

[12] Department of Neurosurgery, Lancashire Teaching Hospitals NHS Foundation Trust, Preston, PR2 9HT

[13] Department of Neurosurgery, Cambridge University Hospitals NHS Foundation Trust, Cambridge, CB2 0QQ

[14] Department of Neurosurgery, Hull University Teaching Hospitals NHS Trust, Hull, HU3 2JZ

[15] Department of Radiology, Hull University Teaching Hospitals NHS Trust, Hull, HU3 2JZ

[16] Department of Neurosurgery, South Tees Hospitals NHS Foundation Trust, Middlesbrough, TS4 3BW

[17] Department of Neurosurgery, Northern Care Alliance NHS Foundation Trust, Salford, M6 8HD

[18] Department of Neurosurgery, Oxford University Hospitals NHS Foundation Trust, Oxford, OX3 9DU

[19] Department of Neurosurgery, Newcastle Hospitals NHS Foundation Trust, Newcastle, NE1 4LP

[20] Institute of Neurological Sciences, NHS Greater Glasgow and Clyde, Glasgow, G51 4TF

For peer review only

[21] Department of Neurosurgery, North Bristol NHS Trust, Bristol, BS10 5NB

[22] Department of Neurosurgery, King's College Hospital NHS Foundation Trust, London, SE5 9RS

[23] Department of Neurosurgery, University Hospitals of North Midlands NHS Trust, Stoke-on-Trent, ST4 6QG

[24] Department of Neurosurgery, Barts Health NHS Trust, London, E1 1BB

[25] Department of Renal Medicine, University Hospitals Portsmouth NHS Trust, Portsmouth, PO6 3LY

[26] Department of Neurosurgery, University Hospitals Birmingham NHS Foundation Trust, Birmingham, B15 2WB

[27] South West Neurosurgery Centre, University Hospitals Plymouth NHS Trust, Plymouth PL6 8DH

Corresponding author:

Mr Diederik Bulters
dbulters@nhs.net
Tel: 0044 2381 205311
Department of neurosurgery
Southampton General Hospital
Tremona Road
Southampton
SO16 6YD
United Kingdom

For peer review only

Abstract

Introduction:

Unruptured intracranial aneurysms (UIA) are common in the adult population, but only a relatively small proportion will rupture. It is therefore essential to have accurate estimates of rupture risk to target treatment towards those who stand to benefit and avoid exposing patients to the risks of unnecessary treatment. The best available UIA natural history data is the PHASES study. However, this has never been validated and given the known heterogeneity in the populations, methods and biases of the constituent studies, there is a need to do so. There are also many potential predictors not considered in PHASES that require evaluation, and the estimated rupture risk is largely based on short term follow up (mostly 1 year). This study's aims are: 1) test the accuracy of PHASES in a UK population, 2) evaluate additional predictors of rupture and 3) assess long-term UIA rupture rates.

Methods and analysis:

The Risk Of Aneurysm Rupture Study is a longitudinal multicentre study that will identify patients with known UIA seen in neurosurgery units. Patients will have baseline demographics and aneurysm characteristics collected by their neurosurgery unit and then a single aggregated national cohort will be linked to databases of hospital admissions and deaths to identify all patients who may have subsequently suffered a subarachnoid haemorrhage. All matched admissions and deaths will be checked against medical records to confirm the diagnosis of aneurysmal subarachnoid haemorrhage. The target sample size is 20,000 patients. The primary outcome will be aneurysm rupture resulting in hospital admission or death. Cox regression models will be built to test each of the study's aims.

Ethics and dissemination:

Ethical approval has been given by South Central Hampshire A REC and Confidentiality Advisory Group support provided under Section 251 of the NHS Act 2006. The results will be disseminated in peer reviewed journals.

Protocol version: 2.1 12th July 2022

Trial registration number: ISRCTN 17658526. Date of registration: 21/4/2021.

Key words: intracranial aneurysm, aneurysm cerebral, rupture risk, aneurysmal subarachnoid haemorrhage, natural history, survival analysis, validation study.

Strengths and limitations of this study

- This study will validate the PHASES score for UIA rupture risk prediction in a population relevant to the UK.
- The UIA treatment rate is lower in the UK than in many other developed countries, which reduces selection bias and allows observation of the true UIA natural history.
- The large cohort size will allow inclusion of more covariates in prediction models than has been possible in previous studies.
- This study will include rare, but salient, patient groups such as those with Autosomal Dominant Polycystic Kidney Disease (ADPKD).

For peer review only

- The UK has uniform medical coverage and given virtually all patients who suffer subarachnoid haemorrhage seek medical help, the strategy to use national databases for hospital admissions and deaths in a defined population provides a robust method for identification of outcome events with minimal loss to follow up.
- This design makes ROAR not only an order of magnitude larger than previous natural history studies but allows for repeated follow up and generation of true long-term rupture risk.

Introduction

Unruptured intracranial aneurysms (UIA) are common in the general population with an estimated prevalence of 2.3-3.2%.[1,2] Aneurysm rupture resulting in subarachnoid haemorrhage is much less common with an annual incidence of 9 per 100,000 of the population.[3] It is estimated that 1.4% of UIA rupture per year.[4] Subarachnoid haemorrhage is a serious complication of UIA with a mortality rate of up to 67% and half of the survivors are left disabled.[5] Unruptured intracranial aneurysms can be prophylactically treated to prevent rupture, however these procedures carry at least a 5% risk of complications.[6] In the absence of randomised controlled trial data, the decision on proceeding to prophylactic treatment is dependent on natural history data. Decisions regarding whether to follow up untreated patients radiologically also depend on our understanding of this data.

The first natural history study, and the most applicable to the UK population, was the International Study of Unruptured Intracranial Aneurysms (ISUIA).[7] However, concerns over the data are well documented,[8] and the generalisability of the results may be undermined by selection bias resulting from a high treatment rate of 71%. Five further natural history studies have been conducted,[9-13] all in different populations with different selection biases and different periods of follow up, and yielding different results. For example, Juvela *et al.*[9] reported rupture rates of 26% of UIA<7mm over 30 years compared to 0% in similar aneurysms extrapolated from ISUIA. This difference may reflect a higher risk in the Finnish population or difference in study methodology – it is not known which.

These six studies were combined in an individual patient level meta-analysis as the PHASES score which provides an estimate for 5 year rupture risk.[4] The PHASES score is the best available evidence for UIA rupture risk. However, it has never been externally validated, and particularly given the heterogeneity in the underlying studies, there is an urgent need to do so.

Furthermore, PHASES was limited to the risk factors available for analysis from the underlying studies. There are many more patient and aneurysm features which have been shown to be associated with rupture or that may be hypothesised to predispose to rupture. These range from common modifiable variables like smoking, to rarer non-modifiable ones like family history and ADPKD.

One of the main shortfalls of PHASES is that, with one exception, the constituent studies are based on short lengths of follow-up, with the majority of patients followed up just 1 year, which has been used to generate 5 year risks in PHASES. Clinicians further extrapolate PHASES to patient’s lifetime risk which all makes the large assumption that risk does not change over time. Moreover, even if the

For peer review only

bleeding risk remains constant over time, any seemingly small inaccuracies in short term estimates can become very significant when extrapolated over many decades.

We therefore designed a large multicentre longitudinal study of patients with UIAs to address these concerns.

Methods and analysis

Objectives

This study has three objectives:

1) To measure the accuracy of the PHASES score at predicting UIA rupture rates in the UK population.
2) To develop a new, more personalised, predictive model for aneurysm rupture incorporating additional co-variates thought to influence risk.
3) To measure aneurysm rupture risk over time periods greater than 5 years.

Study Setting

This is a multicentre study conducted at up to 30 tertiary neurosurgery units in the United Kingdom. Patients will be identified by the neurosurgery unit who diagnosed their unruptured intracranial aneurysm. Each unit will collect their baseline data on patients' clinical and aneurysm characteristics from the time of diagnosis. Central searches of hospital admissions databases, and data analysis, will be performed by the co-ordinating team at University Hospital Southampton NHS Foundation Trust and the University of Oxford. A separate cohort enriched in patients with autosomal dominant polycystic kidney disease will be established using similar methodology, from up to 70 renal units in the United Kingdom.

Study design

The ROAR study is a longitudinal study that uses a hybrid design of patient identification at regional neurosurgical units and prospectively collected national hospital admissions databases for outcome events. The study will establish a cohort of patients with a UIA and measure how many subsequently ruptured. This observed rupture rate can be compared to a rate estimated by the PHASES score to determine its accuracy.

Each neurosurgery unit in the UK will be invited to search their medical records for patients diagnosed with a UIA. This search method will be tailored by the individual neurosurgery unit based on what records they keep, but search strategies may include: Multi-Disciplinary Team (MDT) meeting logs, radiology reports or electronic patient records. The search strategy will be predefined by individual units dependent on their record systems. The maximum date range for identifying patients is documents dated 1/1/2006-31/12/2020, however, this period may be shorter for each unit depending on availability of records. Whatever date range is chosen by the unit it will be pre-defined and if the patient's UIA was newly diagnosed during this period it will be classed as *new* and

For peer review only

those who were diagnosed before this period but identified from a document during the aneurysm follow-up will be classed as *follow-up*, and their recruitment date recorded as the date of the document from which they were identified. This will minimise the prevalence-incidence (Neyman)[14] selection bias created by identifying patients diagnosed before the unit's search period but who survive without rupture to make it into the search period whereas their counterparts diagnosed at the same time who rupture and die are not identified. It will also allow comparison of rupture risk of newly diagnosed aneurysms and those with known diagnoses. Baseline clinical characteristics and aneurysm characteristics will be collected as per the common data elements for UIA research.[15] All data collectors will undergo training in coding data elements delivered by the co-ordinating centre. Collecting baseline data from local medical records allows deeper phenotypic typing and higher data fidelity than using national admission databases.

Patient identifiable details (name, date of birth, post code, NHS/CHI number) will be securely sent by each neurosurgery unit to the co-ordinating centre for consolidation and linkage to the national databases for hospital admissions. These databases are: Hospital Episode Statistics (HES), Patient Episode Database for Wales (PEDW) and Scottish Morbidity Database (SMD). These databases also link to the Civil Registrations - Death and National Registry Scotland for death records. Patients will be linked to hospital admissions based on ICD10 diagnosis codes for intracranial haemorrhages and OPCS4 codes for aneurysm occlusion treatments. These databases record every hospital admission in their respective country and thus using this as the outcome source, combined with death records, will allow identification of every aneurysmal subarachnoid haemorrhage (aSAH) regardless of whether they were managed in a neurosurgery unit, a district general hospital, migrated out of the region in which their UIA was diagnosed, or died in the community. The number of patients with aSAH who are not captured by this method, either because they do not present to hospital or emigrate out of the UK, is expected to be very small. Rupture rates can be adjusted based on national emigration rates.

Patients will be censored if there is any occlusive treatment of the unruptured intracranial aneurysm or patient death. Occlusive treatment includes either microsurgical or endovascular techniques either partial or complete. If none of these censoring events are observed, then they will be censored on the day the cohort is submitted to the HES/PEDW/SMD databases.

The ICD10 codes to be searched for rupture events have been selected in accordance with the UK Biobank stroke research and include: aneurysmal subarachnoid haemorrhage (I60.1-9), intra-cerebral haemorrhage (I61.0-9), traumatic subarachnoid haemorrhage (S06.6) and spontaneous subdural haemorrhage (I62.00-I62.02).[16] The use of codes beyond those for aneurysmal subarachnoid haemorrhage will capture any hospital admissions for aneurysm rupture which have resulted in other forms of intracranial bleed or have been mis-coded.

It is expected that the use of codes beyond that for just aSAH will return many admissions not due to aneurysm rupture. The matched hospital admissions, and death records, will be returned to the co-ordinating unit who will in turn use pseudonymisation numbers to inform the respective local unit of their patient's admission for possible aneurysmal subarachnoid haemorrhage. The local unit will review the imaging studies, discharge summaries and death certificates for these admissions and

For peer review only

confirm or refute the diagnosis. Statistical analysis will begin once the diagnosis for all of the matched admissions is confirmed.

Eligibility criteria

Inclusion:

1. Age 18 years or older.
2. Intracranial, intradural, unruptured aneurysm.
3. Aneurysm confirmed on cranial angiogram (CTA/MRA/DSA).
4. Identification of UIA from records between 1st January 2006 - 31st December 2020.

Exclusion:

1. Mycotic or vasculitic aneurysms.
2. Aneurysm diagnosed on CT or MRI alone.
3. AVM associated flow aneurysms.
4. Extradural aneurysms (e.g. intra-cavernous).
5. Aneurysms treated by either microsurgical or endovascular techniques before the search period.
6. Small lesions uncertain as to whether they are truly aneurysmal ("dilatation", "bulge", 'Infundibulum").

Outcomes

- Primary endpoints

The primary endpoint is rupture of an untreated unruptured intracranial aneurysm at a timepoint at least one day following diagnosis. A rupture event is defined as either radiological evidence of aneurysmal subarachnoid haemorrhage in a distribution consistent with the aneurysm location, CSF spectrophotometry positive for xanthochromia per the local unit's reference range, or death certificate stating subarachnoid haemorrhage in either 1a-c.

- Secondary endpoints

The secondary endpoint is aneurysm growth on follow up imaging. Recruiting units will record if patients have undergone follow up imaging. Aneurysm growth will be recorded if there was any clinically observable growth, in the opinion of a consultant neuroradiologist or an MDT, when directly comparing baseline and follow up scans.

Data transmission and editing

The recruiting units will populate two data sheets, one containing patient identifiable details required for hospital admission database searches and a second containing clinical details only. These two spreadsheets will be cross-referenced using an aneurysm-level pseudonymisation number contained in both spreadsheets. Recruiting units will send each data sheet to the co-ordinating unit separately through a 256bit end to end encryption service.

For peer review only

The requirement for editing the data will be minimised through the use of restricted fields and pre-defined lists of valid codes for each element on the datasheet. All queries and discrepancies raised by the co-ordinating centre regarding the data entry will be submitted to the respective recruiting units through a single query sheet referencing the pseudonymisation number.

Sample size

There are no accepted methods for power calculation for validation studies of prognostic models. Earlier methods included the rule of thumb to have ten events for every covariate tested, however, more modern methods for minimum sample size calculation have been proposed by Riley *et al.*[17] The online package *pmsampsize* uses the Riley method to estimate the minimum sample size. Using figures from our feasibility work (2,124 patients with 60 rupture events over 4,010 patient years), estimating 28 degrees of freedom, and varying Cox-Snell R^2 value d from 0.03 to 0.05 resulted in a minimum sample size ranging from 5,143 to 8,559. The number of degrees of freedom allows for all categorical variables as well as continuous variables such as patient age or aneurysm size which may require polynomial equations.

The Riley method is yet to be widely utilized and does not consider the prevalence of uncommon variables such as ADPKD. The older rule of thumb requiring 10 events per covariate was therefore also considered. For the first objective, the 6 covariates in the PHASES score will be tested suggesting at least 60 SAHs will need to be captured. ISUIA recorded 51 ruptures in 1,692 patients over 4.1 years. Sixty events may therefore be expected in 1,990 patients with 8,161 years of follow up. For the second objective, 120 events will need to be observed to account for the 6 additional commonly occurring covariates which would be expected in 3,981 patients with 16,332 years of follow up.

However, the 10 events per covariate rule of thumb does not consider the prevalence of the covariate in the study population. Therefore, rarely occurring populations may have insufficient data to estimate risk. ADPKD is one such population and including it as a covariate requires a larger study size. In ADPKD 10 events are expected in 1,360 patient years of follow up assuming the risk of SAH is similar to the general population. However, 16,332 years follow up would only yield 195 years in patients with ADPKD (based on a population study which found 53/4,436 patients with UIA had ADPKD).[18] Therefore 113,905 years of follow up would be required to capture 10 ruptures amongst 1,340 years follow up in ADPKD patients. This equates to a total cohort of 22,781 patients.

The TRIPOD guidelines highlight the lack of consensus of how to calculate a sample size and suggest aiming for larger sample sizes which give more precise and reliable results. Smaller sample sizes are at risk of performance optimism. Therefore, the ROAR study will aim to collect 20,000 patients. Based on feasibility studies, this is the maximum practical sample size, and power calculations show is sufficient to generate precise estimates and account for all covariates.

Statistical analysis

- *Objective 1 - PHASES validation*

The PHASES study provides the coefficients from their Cox regression model and baseline survival at 5 years which allows the absolute 5-year risk of rupture to be calculated for all patients who are not censored before 5 years. Time to censoring will be calculated (whichever is soonest of the date of treatment, date of death or the HES/PEDW/SMD search date) to ensure 5 years of follow up if

For peer review only

rupture has not occurred. Discrimination will be assessed using Harrell's C-index of concordance and Royston and Sauerbrei's D statistic. Calibration will be assessed at the 5 year time point using the method in Royston (2014).[19] These will be used to calculate the number of SAH events per 5 years for each PHASES score (≤2 to 12+) and expressed as a percentage with 95% confidence interval to compare to the PHASES estimates.

- *Objective 2 - Additional prognostic factors*

A new risk prediction model for rupture will be developed using the total data set, including the additional possible risk factors. The Cox regression model will be used for risk of rupture. The absolute risks can be estimated at relevant time intervals, 2, 5 and 10 years. Numbers of missing values will be summarised for each factor. Multiple imputation will be used to replace missing values. Discrimination of the final model will be assessed with Harrell's C-statistic. Internal validity will be assessed by bootstrap resampling.

- *Objective 3 – Long term rupture rates*

All patients, including those who underwent aneurysm occlusion, will be included in time-to-event analysis which will cover the whole duration of available follow up. This will include Kaplan-Meier and proportional hazards models for univariate and multivariate survival curve fitting. A cumulative Hazard plot will be used to assess if rupture risk is constant or varies with time from diagnosis.

Once the cohort is established, funding will be sought for repeated searches at regular intervals (5 yearly) to update models and provide progressively longer-term rupture rates.

Patient and public involvement

A workgroup was organised with the Wessex Subarachnoid Haemorrhage Support Group to discuss UIA research where it was confirmed that better decision making on aneurysm treatment is the main concern for patients, but patients do not want to have their management randomised and therefore an RCT is unlikely to succeed. Consequently, a better understanding of the natural history of UIA was deemed the top priority and that long term, ideally lifetime risks, are what is relevant to patients. This formed the basis of the current study.

During May 2020, while face to face public involvement was not possible due to the COVID pandemic, patients in the neurovascular telephone clinic at University Hospital Southampton were surveyed to assess the study design. All patients strongly supported a study of the natural history of UIA. Although some said they would decline participation in imaging or interventional studies, all confirmed they would be happy for their records to be searched for a natural history study, without full informed consent as is proposed.

Ethical considerations (including informed consent)

Seeking informed consent from all patients to break confidentiality and transfer their identifiable details is not possible without biasing the results. Patients whose aneurysm ruptures have a high likelihood of death or severe disability which would leave them unable to provide informed consent.

For peer review only

If informed consent was mandatory, the final cohort would contain an underrepresentation of patients whose aneurysm ruptured, thus skewing the observed rupture rates.

In order to process patient identifiable data without consent the study has been given conditional support under Section 251 from the HRA Confidentiality Advisory Group (21/CAG/0033). This allows the transfer of patient identifiable data outside of the direct clinical care team for the purpose of this study. The patient identifiable data can thus be transferred to the co-ordinating team who in turn can upload this data to the HES/PEDW/SMD databases. The protocol has also been reviewed by the South Central Hampshire A Research Ethics Committee and issued a favourable opinion in March 2021 (21/SC/0064). The REC and CAG committees will be updated on all significant protocol amendments by the study co-ordinator.

Monitoring

As a study without direct patient contact there will not be a separate data monitoring committee. Instead, this role will be conducted by the trial management committee.

Dissemination and Data availability

The results will be disseminated in peer reviewed journals. Authorship will follow International Committee of Medical Journal Editors recommendations and professional writers will not be used. Upon completion of the study, the anonymised dataset will be available both to members of the ROAR collaboration and other external researchers. They will be available from the chief investigator upon reasonable request.

Discussion

Although the natural history of UIA has been previously investigated through multiple prospective studies, the rupture rate has not yet been reliably established. In the absence of randomised clinical data, the natural history of aneurysm rupture forms a core part of treatment decision making. The PHASES score is currently the best available evidence for estimating rupture risk. However, it has never been validated and is limited to estimating risks for 5 years only. The ROAR study will provide observed rupture rates of UIA in clinical practice in the UK which will be used to assess the accuracy of the PHASES score, build models with additional covariates and observe rupture rates beyond 5 years.

References

1 Vlak, M. H., Algra, A., Brandenburg, R. & Rinkel, G. J. Prevalence of unruptured intracranial aneurysms, with emphasis on sex, age, comorbidity, country, and time period: a systematic review and meta-analysis. *Lancet Neurol* **10**, 626-636, doi:10.1016/S1474-4422(11)70109-0 (2011).

For peer review only

2 Cras, T. Y. *et al.* Determinants of the Presence and Size of Intracranial Aneurysms in the General Population: The Rotterdam Study. *Stroke* **51**, 2103-2110, doi:10.1161/STROKEAHA.120.029296 (2020).
3 de Rooij, N. K., Linn, F. H., van der Plas, J. A., Algra, A. & Rinkel, G. J. Incidence of subarachnoid haemorrhage: a systematic review with emphasis on region, age, gender and time trends. *J Neurol Neurosurg Psychiatry* **78**, 1365-1372, doi:10.1136/jnnp.2007.117655 (2007).
4 Greving, J. P. *et al.* Development of the PHASES score for prediction of risk of rupture of intracranial aneurysms: a pooled analysis of six prospective cohort studies. *Lancet Neurol* **13**, 59-66, doi:10.1016/S1474-4422(13)70263-1 (2014).
5 Nieuwkamp, D. J. *et al.* Changes in case fatality of aneurysmal subarachnoid haemorrhage over time, according to age, sex, and region: a meta-analysis. *Lancet Neurol* **8**, 635-642, doi:10.1016/S1474-4422(09)70126-7 (2009).
6 Algra, A. M. *et al.* Procedural Clinical Complications, Case-Fatality Risks, and Risk Factors in Endovascular and Neurosurgical Treatment of Unruptured Intracranial Aneurysms: A Systematic Review and Meta-analysis. *JAMA Neurol* **76**, 282-293, doi:10.1001/jamaneurol.2018.4165 (2019).
7 Wiebers, D. O. *et al.* Unruptured intracranial aneurysms: natural history, clinical outcome, and risks of surgical and endovascular treatment. *Lancet* **362**, 103-110 (2003).
8 Raymond, J. *et al.* Unruptured Intracranial Aneurysms. A Critical Review of the International Study of Unruptured Intracranial Aneurysms (ISUIA) and of Appropriate Methods to Address the Clinical Problem. *Interv Neuroradiol* **14**, 85-96, doi:10.1177/159101990801400111 (2008).
9 Juvela, S., Poussa, K., Lehto, H. & Porras, M. Natural history of unruptured intracranial aneurysms: a long-term follow-up study. *Stroke* **44**, 2414-2421, doi:10.1161/STROKEAHA.113.001838 (2013).
10 Morita, A. *et al.* The natural course of unruptured cerebral aneurysms in a Japanese cohort. *N Engl J Med* **366**, 2474-2482, doi:10.1056/NEJMoa1113260 (2012).
11 Wermer, M. J. *et al.* Yield of short-term follow-up CT/MR angiography for small aneurysms detected at screening. *Stroke* **37**, 414-418, doi:10.1161/01.STR.0000199077.06390.35 (2006).
12 Sonobe, M., Yamazaki, T., Yonekura, M. & Kikuchi, H. Small unruptured intracranial aneurysm verification study: SUAVe study, Japan. *Stroke* **41**, 1969-1977, doi:10.1161/STROKEAHA.110.585059 (2010).
13 Ishibashi, T. *et al.* Unruptured intracranial aneurysms: incidence of rupture and risk factors. *Stroke* **40**, 313-316, doi:10.1161/STROKEAHA.108.521674 (2009).
14 Delgado-Rodríguez, M. & Llorca, J. Bias. *J Epidemiol Community Health* **58**, 635-641, doi:10.1136/jech.2003.008466 (2004).
15 Hackenberg, K. A. M. *et al.* Definition and Prioritization of Data Elements for Cohort Studies and Clinical Trials on Patients with Unruptured Intracranial Aneurysms: Proposal of a Multidisciplinary Research Group. *Neurocrit Care* **30**, 87-101, doi:10.1007/s12028-019-00729-0 (2019).
16 Woodfield, R., Grant, I., Sudlow, C. L., Group, U. B. S. O. & Group, U. B. F.-U. a. O. W. Accuracy of Electronic Health Record Data for Identifying Stroke Cases in Large-Scale Epidemiological Studies: A Systematic Review from the UK Biobank Stroke Outcomes Group. *PLoS One* **10**, e0140533, doi:10.1371/journal.pone.0140533 (2015).
17 Riley, R. D. *et al.* Calculating the sample size required for developing a clinical prediction model. *BMJ* **368**, m441, doi:10.1136/bmj.m441 (2020).
18 Nurmonen, H. J. *et al.* Polycystic kidney disease among 4,436 intracranial aneurysm patients from a defined population. *Neurology* **89**, 1852-1859, doi:10.1212/WNL.0000000000004597 (2017).

For peer review only

19 Royston, P. Tools for Checking Calibration of a Cox Model in External Validation: Approach Based on Individual Event Probabilities. *The Stata Journal* **14**, 738-755, doi:10.1177/1536867x1401400403 (2014).

Authors' contributions

DB conceived the study. DB, SH, and JB designed the protocol. JB provided statistical support and advised on study design. PG, DD and CH helped piloting and testing the protocol. CT, MT, JP, CU, HP, NG, IA, NT, JvB and JG provided further feedback on the protocol. All remaining authors contributed to the set-up of the study, have reviewed the protocol and approved the final manuscript.

Conflict of interest

None of the authors have any conflicts to declare.

Sponsor contact information

Sponsor: University Hospital Southampton NHS Foundation Trust

Sponsor reference: RHM NEU0390

Contact name: Mrs Sharon Davies-Dear

Contact email: Sharon.davies-dear@uhs.nhs.uk

Sponsor and funder involvement

This study/project is funded by the National Institute for Health Research (NIHR) Research for Patient Benefit programme (NIHR203628). The views expressed are those of the author(s) and not necessarily those of the NIHR or the Department of Health and Social Care.

This work is also supported by: Smile4Wessex (no grant number) and the Polycystic Kidney Disease Charity (PKD-21-03). Neither the sponsor nor any of the funding sources had a role in designing the study protocol. They will also have no role in the analysis of data nor submission of results.

ROAR Collaborators

Frederick Ewbank,[1] Stefan Mitrasinovic,[1] Oliver Croft,[1] Dominic Townsend[1]

Neeraj Kalra,[3] Imogen Kirkpatrick,[3] Nicole Handy[3]

Alex Rossdeutsch,[4] Ellie Courtney,[4] Zoe Stone[4]

Jonny Downer,[5] James Loan,[5] Anthony Wiggins,[5] Jay Park,[5] Niamh Rafferty[5]

Nathan McSorley,[6] Ajitesh Anand,[6] Armin Nazari[6]

For peer review only

Giannis Sokratous,[7] Basel Taweel,[7] Kartik Goyal[7]

John Norris,[8] Hannah-Marie Culley[8], Mukul Arora[8]

Milo Hollingworth,[9] Megan Burns,[9] Benjamin Armstrong[9]

Janneke van Beijnum,[10] Harsh Bhatt,[10] David Lowes,[10] Debra Faulkner[10]

Ciaran Hill,[11] Debayan Dasgupta,[11] Shuja Yaqub,[11] Yuzhi Phuah,[11] Olivier Sluijters,[11] Neda Oskooee,[11] Viraj Pamar,[11] Tarek Elmenofi,[11] Justyna Ekert[11]

Alex Fung,[13] Rebecca Legge[13]

Srihari Deepak,[15] Vigneshwar Veerappan,[14] Swarnava Gupta,[14] Sumeet Sasane,[14] May Ting Tan[15]

Lucie Ferguson[16]

Matthew Myers,[4,17] Antonio Bonardi,[17] Holly Tetlow,[17] Charlotte Dunkerley,[17] Malvika Pandey,[17] William Giffin,[17] Iqbal Bin Lokman,[17] Danielle Hurst,[17] Daniel Ahari[17]

Anouk Borg,[18] Thanos Papadias[18]

Kristy Kehoe,[19] Jeremy Cheong,[19] Isabella Davies,[19] Taisha Peplowska[19]

James Ulrich[20] Mohammed Draz,[20] Barbora Krivankova,[20] Katy Homyer,[20] Vivienne Evans,[20] Steven Tominey,[20] Attika Chaudhary,[20] Sabrina Tengku,[20] Eilidh Middleton,[20] Sytske Lub,[20] Hassan Ismahel,[20] Michalina Wilinska,[20] Nicola Duncan,[20] Joanne Igoli,[20] Ritika Sandaram[20]

Rebecca Hodnett,[21] Naadir Nazar,[21] Omar Ouaret Sorr,[21] Fatima Camp,[21] Tom Ferreira[21]

Benjamin Fisher,[22] Devika Rajashekar,[22] Rachel Dumbrell,[22] Ananya Muthukumar,[22] Mohammad Anas,[22] Lilian Nwosu,[22] Ariadne Holmes[22]

Ikenna Ogbu,[23] Madalina Pasca,[23] Veer Patel,[23] Mustafa Dashti,[23] Zeluleko Sibanda,[23] Erin Hwang,[23] Zara Adil[23]

Samir Matloob,[24] Vanessa Chow[24]

Rosa Sun,[26] Aparnu Vimal,[26] Talhah Chaudri,[26] Lauren Baldwin[26]

Haritha Maripi,[27] Balint Borbas,[27] Mariyam Mujeeb[27]

For peer review only

For peer review only

STANDARD PROTOCOL ITEMS: RECOMMENDATIONS FOR INTERVENTIONAL TRIALS

SPIRIT 2013 Checklist: Recommended items to address in a clinical trial protocol and related documents*

Section/item	ItemNo	Description	Reported on page
**Administrative information**			
Title	1	Descriptive title identifying the study design, population, interventions, and, if applicable, trial acronym	1
Trial registration	2a	Trial identifier and registry name. If not yet registered, name of intended registry	3
	2b	All items from the World Health Organization Trial Registration Data Set	3, 12
Protocol version	3	Date and version identifier	3
Funding	4	Sources and types of financial, material, and other support	12
Roles and responsibilities	5a	Names, affiliations, and roles of protocol contributors	12
	5b	Name and contact information for the trial sponsor	12
	5c	Role of study sponsor and funders, if any, in study design; collection, management, analysis, and interpretation of data; writing of the report; and the decision to submit the report for publication, including whether they will have ultimate authority over any of these activities	12
	5d	Composition, roles, and responsibilities of the coordinating centre, steering committee, endpoint adjudication committee, data management team, and other individuals or groups overseeing the trial, if applicable (see Item 21a for data monitoring committee)	N/A
**Introduction**			
Background and rationale	6a	Description of research question and justification for undertaking the trial, including summary of relevant studies (published and unpublished) examining benefits and harms for each intervention	4-5
	6b	Explanation for choice of comparators	N/A
Objectives	7	Specific objectives or hypotheses	5

For peer review only

Trial design	8	Description of trial design including type of trial (eg, parallel group, crossover, factorial, single group), allocation ratio, and framework (eg, superiority, equivalence, noninferiority, exploratory)	5
**Methods: Participants, interventions, and outcomes**			
Study setting	9	Description of study settings (eg, community clinic, academic hospital) and list of countries where data will be collected. Reference to where list of study sites can be obtained	5
Eligibility criteria	10	Inclusion and exclusion criteria for participants. If applicable, eligibility criteria for study centres and individuals who will perform the interventions (eg, surgeons, psychotherapists)	7
Interventions	11a	Interventions for each group with sufficient detail to allow replication, including how and when they will be administered	N/A
	11b	Criteria for discontinuing or modifying allocated interventions for a given trial participant (eg, drug dose change in response to harms, participant request, or improving/worsening disease)	N/A
	11c	Strategies to improve adherence to intervention protocols, and any procedures for monitoring adherence (eg, drug tablet return, laboratory tests)	N/A
	11d	Relevant concomitant care and interventions that are permitted or prohibited during the trial	N/A
Outcomes	12	Primary, secondary, and other outcomes, including the specific measurement variable (eg, systolic blood pressure), analysis metric (eg, change from baseline, final value, time to event), method of aggregation (eg, median, proportion), and time point for each outcome. Explanation of the clinical relevance of chosen efficacy and harm outcomes is strongly recommended	7
Participant timeline	13	Time schedule of enrolment, interventions (including any run-ins and washouts), assessments, and visits for participants. A schematic diagram is highly recommended (see Figure)	N/A
Sample size	14	Estimated number of participants needed to achieve study objectives and how it was determined, including clinical and statistical assumptions supporting any sample size calculations	8
Recruitment	15	Strategies for achieving adequate participant enrolment to reach target sample size	N/A

For peer review only - http://bmjopen.bmj.com/site/about/guidelines.xhtml

**Methods: Assignment of interventions (for controlled trials)**			
Allocation:			
Sequence generation	16a	Method of generating the allocation sequence (eg, computer-generated random numbers), and list of any factors for stratification. To reduce predictability of a random sequence, details of any planned restriction (eg, blocking) should be provided in a separate document that is unavailable to those who enrol participants or assign interventions	N/A
Allocation concealment mechanism	16b	Mechanism of implementing the allocation sequence (eg, central telephone; sequentially numbered, opaque, sealed envelopes), describing any steps to conceal the sequence until interventions are assigned	N/A
Implementation	16c	Who will generate the allocation sequence, who will enrol participants, and who will assign participants to interventions	N/A
Blinding (masking)	17a	Who will be blinded after assignment to interventions (eg, trial participants, care providers, outcome assessors, data analysts), and how	N/A
	17b	If blinded, circumstances under which unblinding is permissible, and procedure for revealing a participant's allocated intervention during the trial	N/A
**Methods: Data collection, management, and analysis**			
Data collection methods	18a	Plans for assessment and collection of outcome, baseline, and other trial data, including any related processes to promote data quality (eg, duplicate measurements, training of assessors) and a description of study instruments (eg, questionnaires, laboratory tests) along with their reliability and validity, if known. Reference to where data collection forms can be found, if not in the protocol	5-7
	18b	Plans to promote participant retention and complete follow-up, including list of any outcome data to be collected for participants who discontinue or deviate from intervention protocols	N/A
Data management	19	Plans for data entry, coding, security, and storage, including any related processes to promote data quality (eg, double data entry; range checks for data values). Reference to where details of data management procedures can be found, if not in the protocol	7
Statistical methods	20a	Statistical methods for analysing primary and secondary outcomes. Reference to where other details of the statistical analysis plan can be found, if not in the protocol	8-9

For peer review only - http://bmjopen.bmj.com/site/about/guidelines.xhtml

	20b	Methods for any additional analyses (eg, subgroup and adjusted analyses)	N/A
	20c	Definition of analysis population relating to protocol non-adherence (eg, as randomised analysis), and any statistical methods to handle missing data (eg, multiple imputation)	8-9
**Methods: Monitoring**			
Data monitoring	21a	Composition of data monitoring committee (DMC); summary of its role and reporting structure; statement of whether it is independent from the sponsor and competing interests; and reference to where further details about its charter can be found, if not in the protocol. Alternatively, an explanation of why a DMC is not needed	10
	21b	Description of any interim analyses and stopping guidelines, including who will have access to these interim results and make the final decision to terminate the trial	N/A
Harms	22	Plans for collecting, assessing, reporting, and managing solicited and spontaneously reported adverse events and other unintended effects of trial interventions or trial conduct	N/A
Auditing	23	Frequency and procedures for auditing trial conduct, if any, and whether the process will be independent from investigators and the sponsor	10
**Ethics and dissemination**			
Research ethics approval	24	Plans for seeking research ethics committee/institutional review board (REC/IRB) approval	9-10
Protocol amendments	25	Plans for communicating important protocol modifications (eg, changes to eligibility criteria, outcomes, analyses) to relevant parties (eg, investigators, REC/IRBs, trial participants, trial registries, journals, regulators)	10
Consent or assent	26a	Who will obtain informed consent or assent from potential trial participants or authorised surrogates, and how (see Item 32)	9-10
	26b	Additional consent provisions for collection and use of participant data and biological specimens in ancillary studies, if applicable	9-10
Confidentiality	27	How personal information about potential and enrolled participants will be collected, shared, and maintained in order to protect confidentiality before, during, and after the trial	7, 9-10
Declaration of interests	28	Financial and other competing interests for principal investigators for the overall trial and each study site	12

For peer review only - http://bmjopen.bmj.com/site/about/guidelines.xhtml

Access to data	29	Statement of who will have access to the final trial dataset, and disclosure of contractual agreements that limit such access for investigators	10
Ancillary and post-trial care	30	Provisions, if any, for ancillary and post-trial care, and for compensation to those who suffer harm from trial participation	N/A
Dissemination policy	31a	Plans for investigators and sponsor to communicate trial results to participants, healthcare professionals, the public, and other relevant groups (eg, via publication, reporting in results databases, or other data sharing arrangements), including any publication restrictions	10
	31b	Authorship eligibility guidelines and any intended use of professional writers	10
	31c	Plans, if any, for granting public access to the full protocol, participant-level dataset, and statistical code	N/A
**Appendices**			
Informed consent materials	32	Model consent form and other related documentation given to participants and authorised surrogates	N/A
Biological specimens	33	Plans for collection, laboratory evaluation, and storage of biological specimens for genetic or molecular analysis in the current trial and for future use in ancillary studies, if applicable	N/A

*It is strongly recommended that this checklist be read in conjunction with the SPIRIT 2013 Explanation & Elaboration for important clarification on the items. Amendments to the protocol should be tracked and dated. The SPIRIT checklist is copyrighted by the SPIRIT Group under the Creative Commons "Attribution-NonCommercial-NoDerivs 3.0 Unported" license.

For peer review only

BMJ Open

Risk Of Aneurysm Rupture (ROAR) study: protocol for a long-term, longitudinal, UK multicentre study of unruptured intracranial aneurysms

Journal:	*BMJ Open*
Manuscript ID	bmjopen-2022-070504.R1
Article Type:	Protocol
Date Submitted by the Author:	24-Feb-2023

Complete List of Authors:	Hall, Samuel; University Hospital Southampton NHS Foundation Trust, Department of Neurosurgery<br>Birks, Jacqueline; University of Oxford, Centre for Statistics in Medicine<br>Anderson, Ian; The Leeds Teaching Hospitals NHS Trust, Department of Neurosurgery<br>Bacon, Andrew; Sheffield Teaching Hospitals NHS Foundation Trust, Department of Neurosurgery<br>Brennan, Paul; Western General Hospital, Department of Clinical Neurosciences<br>Bennett, David; NHS Tayside, Department of Neurosurgery<br>Chavredakis, Emmanuel; Walton Centre NHS Foundation Trust, Department of Neurosurgery<br>Critchley, Giles; University Hospitals Sussex NHS Foundation Trust, Department of Neurosurgery<br>Dow, Graham; Nottingham University Hospitals NHS Trust, Department of Neurosurgery<br>Downer, Jonathan; Western General Hospital, Department of Clinical Neurosciences<br>Galea, James; University Hospital of Wales Healthcare NHS Trust, Neurosurgical Department<br>Grover, Patrick; University College London Hospitals NHS Foundation Trust, National Hospital for Neurology and Neurosurgery<br>Gurusinghe, Nihal; Lancashire Teaching Hospitals NHS Foundation Trust, Department of Neurosurgery<br>Helmy, Adel; Cambridge University Hospitals NHS Foundation Trust, Department of neurosurgery<br>Kounin, Gueorgui; Hull University Teaching Hospitals NHS Trust, Department of Neurosurgery<br>Mukerji, Nitin; South Tees Hospitals NHS Foundation Trust, Department of Neurosurgery<br>Patel, Hiren; Northern Care Alliance NHS Foundation Trust, Department of Neurosurgery<br>Patel, Jash; Oxford University Hospitals NHS Foundation Trust, Department of Neurosurgery<br>Ross, Nicholas; Newcastle Hospitals NHS Foundation Trust, Department of Neurosurgery<br>St George, Jerome; NHS Greater Glasgow and Clyde, Institute of Neurological Sciences<br>Teo, Mario; North Bristol NHS Trust, Department of Neurosurgery

	Tolias, Christos; King's College Hospital NHS Foundation Trust, Neurosurgery<br>Tzerakis, Nikolaos; University Hospitals of North Midlands NHS Trust, Department of Neurosurgery<br>Uff, Christopher; Barts Health NHS Trust, Department of Neurosurgery<br>van Beijum, Janneke; University Hospital of Wales Healthcare NHS Trust, Neurosurgical department<br>Veighey, Kristin; University Hospital Southampton NHS Foundation Trust, Department of Renal Medicine<br>White, Edward; University Hospitals Birmingham NHS Foundation Trust, Department of Neurosurgery<br>Whitfield, Peter; University Hospitals Plymouth NHS Trust, South West Neurosurgery Centre<br>Bulters, Diederik ; University Hospital Southampton NHS Foundation Trust, Department of Neurosurgery<br>ROAR Investigators, ROAR Investigators; University Hospital Southampton NHS Foundation Trust

**Primary Subject Heading**:	Neurology
Secondary Subject Heading:	Evidence based practice, Surgery
Keywords:	EPIDEMIOLOGY, Stroke < NEUROLOGY, NEUROSURGERY

SCHOLARONE™
Manuscripts

Risk Of Aneurysm Rupture (ROAR) study: protocol for a long-term, longitudinal, UK multicentre study of unruptured intracranial aneurysms

Samuel Hall[1], Jacqueline Birks[2], Ian Anderson,[3] Andrew Bacon,[4] Paul Brennan,[5] David Bennett,[6] Emmanuel Chavredakis,[7] Giles Critchley,[8] Graham Dow,[9] Jonny Downer,[5] James Galea,[10] Patrick Grover,[11] Nihal Gurusinghe,[12] Adel Helmy,[13] Gueorgui Kounin,[14] Nitin Mukerji,[16] Hiren Patel,[17] Jash Patel,[18] Nicholas Ross,[19] Jerome St George,[20] Mario Teo,[21] Christos Tolias,[22] Nikolaos Tzerakis,[23] Christopher Uff,[24] Janneke van Beijum,[10] Kristin Veighey,[25] Edward White,[26] Peter Whitfield,[27] Diederik Bulters[1] ROAR Investigators, ROAR Investigators

[1] Department of Neurosurgery, University Hospitals Southampton NHS Foundation Trust, Southampton, UK

[2] Centre for Statistics in Medicine, Medical Sciences Division, University of Oxford, Oxford, UK

[3] Department of Neurosurgery, The Leeds Teaching Hospitals NHS Trust, Leeds, UK

[4] Sheffield Teaching Hospitals NHS Foundation Trust, Sheffield, UK

[5] Department of Clinical Neurosciences, NHS Lothian, Edinburgh, UK

[6] Department of Neurosurgery, NHS Tayside, Dundee, UK

[7] Department of Neurosurgery, Walton Centre NHS Foundation Trust, Liverpool, UK

[8] Department of Neurosurgery, University Hospitals Sussex NHS Foundation Trust, Brighton, UK

[9] Department of Neurosurgery, Nottingham University Hospitals NHS Trust, Nottingham, UK

[10] Neurosurgical Department, Floor 4, Corridor B, University Hospital Wales, Heath Park, Cardiff, UK

[11] National Hospital for Neurology and Neurosurgery, University College London Hospitals NHS Foundation Trust, London, UK

[12] Department of Neurosurgery, Lancashire Teaching Hospitals NHS Foundation Trust, Preston, UK

[13] Department of Neurosurgery, Cambridge University Hospitals NHS Foundation Trust, Cambridge, UK

[14] Department of Neurosurgery, Hull University Teaching Hospitals NHS Trust, Hull, UK

[15] Department of Radiology, Hull University Teaching Hospitals NHS Trust, Hull, UK

[16] Department of Neurosurgery, South Tees Hospitals NHS Foundation Trust, Middlesbrough, UK

[17] Department of Neurosurgery, Northern Care Alliance NHS Foundation Trust, Salford, UK

[18] Department of Neurosurgery, Oxford University Hospitals NHS Foundation Trust, Oxford, UK

[19] Department of Neurosurgery, Newcastle Hospitals NHS Foundation Trust, Newcastle, UK

[20] Institute of Neurological Sciences, NHS Greater Glasgow and Clyde, Glasgow, UK

[21] Department of Neurosurgery, North Bristol NHS Trust, Bristol, UK

For peer review only

[22] Department of Neurosurgery, King's College Hospital NHS Foundation Trust, London, UK

[23] Department of Neurosurgery, University Hospitals of North Midlands NHS Trust, Stoke-on-Trent, UK

[24] Department of Neurosurgery, Barts Health NHS Trust, London, UK

[25] Department of Renal Medicine, University Hospitals Portsmouth NHS Trust, Portsmouth, UK

[26] Department of Neurosurgery, University Hospitals Birmingham NHS Foundation Trust, Birmingham, UK

[27] South West Neurosurgery Centre, University Hospitals Plymouth NHS Trust, Plymouth, UK

Correspondence to:

Mr Diederik Bulters
Department of neurosurgery
Southampton General Hospital
Tremona Road
Southampton
SO16 6YD
UK
dbulters@nhs.net
Tel: 0044 2381 205311

For peer review only

Abstract

Introduction

Unruptured intracranial aneurysms (UIA) are common in the adult population, but only a relatively small proportion will rupture. It is therefore essential to have accurate estimates of rupture risk to target treatment towards those who stand to benefit and avoid exposing patients to the risks of unnecessary treatment. The best available UIA natural history data is the PHASES study. However, this has never been validated and given the known heterogeneity in the populations, methods and biases of the constituent studies, there is a need to do so. There are also many potential predictors not considered in PHASES that require evaluation, and the estimated rupture risk is largely based on short term follow up (mostly 1 year). This study's aims are: 1) test the accuracy of PHASES in a UK population, 2) evaluate additional predictors of rupture and 3) assess long-term UIA rupture rates.

Methods and analysis

The Risk Of Aneurysm Rupture (ROAR) study is a longitudinal multicentre study that will identify patients with known UIA seen in neurosurgery units. Patients will have baseline demographics and aneurysm characteristics collected by their neurosurgery unit and then a single aggregated national cohort will be linked to databases of hospital admissions and deaths to identify all patients who may have subsequently suffered a subarachnoid haemorrhage. All matched admissions and deaths will be checked against medical records to confirm the diagnosis of aneurysmal subarachnoid haemorrhage. The target sample size is 20,000 patients. The primary outcome will be aneurysm rupture resulting in hospital admission or death. Cox regression models will be built to test each of the study's aims.

Ethics and dissemination

Ethical approval has been given by South Central Hampshire A REC (21SC0064) and Confidentiality Advisory Group support (21CAG0033) provided under Section 251 of the NHS Act 2006. The results will be disseminated in peer reviewed journals.

Study registration

ISRCTN17658526 (date of registration: 21/4/2021).

Protocol version: 2.1 12th July 2022

Keywords: intracranial aneurysm, aneurysm cerebral, rupture risk, aneurysmal subarachnoid haemorrhage, natural history, survival analysis, validation study.

Strengths and limitations of this study

- The unruptured intracranial aneurysms (UIA) treatment rate is lower in the UK than in many other developed countries, which reduces selection bias and allows observation of the true UIA natural history.

For peer review only

- The large cohort size will allow inclusion of more covariates in prediction models than has been possible in previous studies including rare, but salient, patient groups such as those with Autosomal Dominant Polycystic Kidney Disease (ADPKD).
- The UK has uniform medical coverage and given virtually all patients who suffer subarachnoid haemorrhage seek medical help, the strategy to use national databases for hospital admissions and deaths in a defined population provides a robust method for identification of outcome events with minimal loss to follow up.
- This design makes the ROAR study not only an order of magnitude larger than previous natural history studies but allows for repeated follow up and generation of true long-term rupture risk.
- Identifying rupture events from hospital admissions databases is reliant on diagnosis coding accuracy; this limitation has been mitigated by searching a broad range of possible codes for intra-cranial haemorrhagic events and subsequent diagnosis verification against original imaging studies.

Introduction

Unruptured intracranial aneurysms (UIA) are common in the general population with an estimated prevalence of 2.3-3.2%.[1,2] Aneurysm rupture resulting in subarachnoid haemorrhage is much less common with an annual incidence of 9 per 100,000 of the population.[3] It is estimated that 1.4% of UIA rupture per year.[4] Subarachnoid haemorrhage is a serious complication of UIA with a mortality rate of up to 67% and half of the survivors are left disabled.[5] Unruptured intracranial aneurysms can be prophylactically treated to prevent rupture, however these procedures carry at least a 5% risk of complications.[6] In the absence of randomised controlled trial data, the decision on proceeding to prophylactic treatment is dependent on natural history data. Decisions regarding whether to follow up untreated patients radiologically also depend on our understanding of this data.

The first natural history study, and the most applicable to the UK population, was the International Study of Unruptured Intracranial Aneurysms (ISUIA).[7] However, concerns over the data are well documented,[8] and the generalisability of the results may be undermined by selection bias resulting from a high treatment rate of 71%. Five further natural history studies have been conducted,[9-13] all in different populations with different selection biases and different periods of follow up, and yielding different results. For example, Juvela *et al.*[9] reported rupture rates of 26% of UIA<7mm over 30 years compared to 0% in similar aneurysms extrapolated from ISUIA. This difference may reflect a higher risk in the Finnish population or difference in study methodology – it is not known which.

These six studies were combined in an individual patient level meta-analysis as the PHASES score which provides an estimate for 5 year rupture risk.[4] The PHASES score is the best available evidence for UIA rupture risk. However, it has never been externally validated, and particularly given the heterogeneity in the underlying studies, there is an urgent need to do so.

Furthermore, PHASES was limited to the risk factors available for analysis from the underlying studies. There are many more patient and aneurysm features which have been shown to be associated with rupture or that may be hypothesised to predispose to rupture. These range from

For peer review only

common modifiable variables like smoking, to rarer non-modifiable ones like family history and ADPKD.

One of the main shortfalls of PHASES is that, with one exception, the constituent studies are based on short lengths of follow-up, with the majority of patients followed up just 1 year, which has been used to generate 5 year risks in PHASES. Clinicians further extrapolate PHASES to patient's lifetime risk which all makes the large assumption that risk does not change over time. Moreover, even if the bleeding risk remains constant over time, any seemingly small inaccuracies in short term estimates can become very significant when extrapolated over many decades.

We therefore designed a large multicentre longitudinal study of patients with UIAs to address these concerns.

Methods and analysis

Objectives

This study has three objectives:

1) To measure the accuracy of the PHASES score at predicting UIA rupture rates in the UK population.
2) To develop a new, more personalised, predictive model for aneurysm rupture incorporating additional co-variates thought to influence risk.
3) To measure aneurysm rupture risk over time periods greater than 5 years.

Study setting

This is a multicentre study conducted at up to 30 tertiary neurosurgery units in the United Kingdom. Patients will be identified by the neurosurgery unit who diagnosed their unruptured intracranial aneurysm. Each unit will collect their baseline data on patients' clinical and aneurysm characteristics from the time of diagnosis. Central searches of hospital admissions databases, and data analysis, will be performed by the co-ordinating team at University Hospital Southampton NHS Foundation Trust and the University of Oxford. A separate cohort enriched in patients with autosomal dominant polycystic kidney disease will be established using similar methodology, from up to 70 renal units in the United Kingdom.

Study design

The Risk Of Aneurysm Rupture (ROAR) study is a longitudinal study that uses a hybrid design of patient identification at regional neurosurgical units and prospectively collected national hospital admissions databases for outcome events. The study will establish a cohort of patients with a UIA and measure how many subsequently ruptured. This observed rupture rate can be compared to a rate estimated by the PHASES score to determine its accuracy.

For peer review only

Each neurosurgery unit in the UK will be invited to search their medical records for patients diagnosed with a UIA. This search method will be tailored by the individual neurosurgery unit based on what records they keep, but search strategies may include: Multi-Disciplinary Team (MDT) meeting logs, radiology reports or electronic patient records. The search strategy will be predefined by individual units dependent on their record systems. The maximum date range for identifying patients is documents dated 1/1/2006-31/12/2020, however, this period may be shorter for each unit depending on availability of records. Whatever date range is chosen by the unit it will be pre-defined and if the patient's UIA was newly diagnosed during this period it will be classed as *new* and those who were diagnosed before this period but identified from a document during the aneurysm follow-up will be classed as *follow-up*, and their recruitment date recorded as the date of the document from which they were identified. This will minimise the prevalence-incidence (Neyman)[14] selection bias created by identifying patients diagnosed before the unit's search period but who survive without rupture to make it into the search period whereas their counterparts diagnosed at the same time who rupture and die are not identified. It will also allow comparison of rupture risk of newly diagnosed aneurysms and those with known diagnoses. Baseline clinical characteristics and aneurysm characteristics will be collected as per the common data elements for UIA research.[15] All data collectors will undergo training in coding data elements delivered by the co-ordinating centre. Collecting baseline data from local medical records allows deeper phenotypic typing and higher data fidelity than using national admission databases.

Patient identifiable details (name, date of birth, post code, NHS/CHI number) will be securely sent by each neurosurgery unit to the co-ordinating centre for consolidation and linkage to the national databases for hospital admissions. These databases are: Hospital Episode Statistics (HES), Patient Episode Database for Wales (PEDW) and Scottish Morbidity Database (SMD). These databases also link to the Civil Registrations - Death and National Registry Scotland for death records. Patients will be linked to hospital admissions based on ICD10 diagnosis codes for intracranial haemorrhages and OPCS4 codes for aneurysm occlusion treatments. These databases record every hospital admission in their respective country and thus using this as the outcome source, combined with death records, will allow identification of every aneurysmal subarachnoid haemorrhage (aSAH) regardless of whether they were managed in a neurosurgery unit, a district general hospital, migrated out of the region in which their UIA was diagnosed, or died in the community. The number of patients with aSAH who are not captured by this method, either because they do not present to hospital or emigrate out of the UK, is expected to be very small. Rupture rates can be adjusted based on national emigration rates.

Patients will be censored if there is any occlusive treatment of the unruptured intracranial aneurysm or patient death. Occlusive treatment includes either microsurgical or endovascular techniques either partial or complete. If none of these censoring events are observed, then they will be censored on the day the cohort is submitted to the HES/PEDW/SMD databases.

The ICD10 codes to be searched for rupture events have been selected in accordance with the UK Biobank stroke research and include: aneurysmal subarachnoid haemorrhage (I60.1-9), intra-cerebral haemorrhage (I61.0-9), traumatic subarachnoid haemorrhage (S06.6) and spontaneous subdural haemorrhage (I62.00-I62.02).[16] The use of codes beyond those for aneurysmal

For peer review only

subarachnoid haemorrhage will capture any hospital admissions for aneurysm rupture which have resulted in other forms of intracranial bleed or have been mis-coded.

It is expected that the use of codes beyond that for just aSAH will return many admissions not due to aneurysm rupture. The matched hospital admissions, and death records, will be returned to the co-ordinating unit who will in turn use pseudonymisation numbers to inform the respective local unit of their patient's admission for possible aneurysmal subarachnoid haemorrhage. The local unit will review the imaging studies, discharge summaries and death certificates for these admissions and confirm or refute the diagnosis. Statistical analysis will begin once the diagnosis for all of the matched admissions is confirmed.

The study is currently opening new sites and identifying patients for inclusion. The baseline data collection is planned to finish by 31/7/2023 and the results released no sooner than 31/7/2024. The study end date is currently 31/7/2034 to allow for repeated searching of hospital admissions databases in the future thereby further extending the follow-up period.

Eligibility criteria

Inclusion:

1. Age 18 years or older.
2. Intracranial, intradural, unruptured aneurysm.
3. Aneurysm confirmed on cranial angiogram (CTA/MRA/DSA).
4. Identification of UIA from records between 1st January 2006 - 31st December 2020.

Exclusion:

1. Mycotic or vasculitic aneurysms.
2. Aneurysm diagnosed on CT or MRI alone.
3. AVM associated flow aneurysms.
4. Extradural aneurysms (e.g. intra-cavernous).
5. Aneurysms treated by either microsurgical or endovascular techniques before the search period.
6. Small lesions uncertain as to whether they are truly aneurysmal ("dilatation", "bulge", 'Infundibulum").

Outcomes

- Primary endpoints

The primary endpoint is rupture of an untreated unruptured intracranial aneurysm at a timepoint at least one day following diagnosis. A rupture event is defined as either radiological evidence of aneurysmal subarachnoid haemorrhage in a distribution consistent with the aneurysm location, CSF spectrophotometry positive for xanthochromia per the local unit's reference range, or death certificate stating subarachnoid haemorrhage in either 1a-c.

For peer review only

- Secondary endpoints

The secondary endpoint is aneurysm growth on follow up imaging. Recruiting units will record if patients have undergone follow up imaging. Aneurysm growth will be recorded if there was any clinically observable growth, in the opinion of a consultant neuroradiologist or an MDT, when directly comparing baseline and follow up scans.

Data transmission and editing

The recruiting units will populate two data sheets, one containing patient identifiable details required for hospital admission database searches and a second containing clinical details only. These two spreadsheets will be cross-referenced using an aneurysm-level pseudonymisation number contained in both spreadsheets. Recruiting units will send each data sheet to the co-ordinating unit separately through a 256bit end to end encryption service.

The requirement for editing the data will be minimised through the use of restricted fields and pre-defined lists of valid codes for each element on the datasheet. All queries and discrepancies raised by the co-ordinating centre regarding the data entry will be submitted to the respective recruiting units through a single query sheet referencing the pseudonymisation number.

Sample size

There are no accepted methods for power calculation for validation studies of prognostic models. Earlier methods included the rule of thumb to have ten events for every covariate tested, however, more modern methods for minimum sample size calculation have been proposed by Riley *et al.*[17] The online package *pmsampsize* uses the Riley method to estimate the minimum sample size. Using figures from our feasibility work (2,124 patients with 60 rupture events over 4,010 patient years), estimating 28 degrees of freedom, and varying Cox-Snell R^2 value d from 0.03 to 0.05 resulted in a minimum sample size ranging from 5,143 to 8,559. The number of degrees of freedom allows for all categorical variables as well as continuous variables such as patient age or aneurysm size which may require polynomial equations.

The Riley method is yet to be widely utilized and does not consider the prevalence of uncommon variables such as ADPKD. The older rule of thumb requiring 10 events per covariate was therefore also considered. For the first objective, the 6 covariates in the PHASES score will be tested suggesting at least 60 SAHs will need to be captured. ISUIA recorded 51 ruptures in 1,692 patients over 4.1 years. Sixty events may therefore be expected in 1,990 patients with 8,161 years of follow up. For the second objective, 120 events will need to be observed to account for the 6 additional commonly occurring covariates which would be expected in 3,981 patients with 16,332 years of follow up.

However, the 10 events per covariate rule of thumb does not consider the prevalence of the covariate in the study population. Therefore, rarely occurring populations may have insufficient data to estimate risk. ADPKD is one such population and including it as a covariate requires a larger study size. In ADPKD 10 events are expected in 1,360 patient years of follow up assuming the risk of SAH is similar to the general population. However, 16,332 years follow up would only yield 195 years in patients with ADPKD (based on a population study which found 53/4,436 patients with UIA had

For peer review only

ADPKD).[18] Therefore 113,905 years of follow up would be required to capture 10 ruptures amongst 1,340 years follow up in ADPKD patients. This equates to a total cohort of 22,781 patients.

The TRIPOD guidelines highlight the lack of consensus of how to calculate a sample size and suggest aiming for larger sample sizes which give more precise and reliable results. Smaller sample sizes are at risk of performance optimism. Therefore, the ROAR study will aim to collect 20,000 patients. Based on feasibility studies, this is the maximum practical sample size, and power calculations show is sufficient to generate precise estimates and account for all covariates.

Statistical analysis

- *Objective 1 - PHASES validation*

The PHASES study provides the coefficients from their Cox regression model and baseline survival at 5 years which allows the absolute 5-year risk of rupture to be calculated for all patients who are not censored before 5 years. Time to censoring will be calculated (whichever is soonest of the date of treatment, date of death or the HES/PEDW/SMD search date) to ensure 5 years of follow up if rupture has not occurred. Discrimination will be assessed using Harrell's C-index of concordance and Royston and Sauerbrei's D statistic. Calibration will be assessed at the 5 year time point using the method in Royston (2014).[19] These will be used to calculate the number of SAH events per 5 years for each PHASES score (≤2 to 12+) and expressed as a percentage with 95% confidence interval to compare to the PHASES estimates.

- *Objective 2 - Additional prognostic factors*

A new risk prediction model for rupture will be developed using the total data set, including the additional possible risk factors. The Cox regression model will be used for risk of rupture. The absolute risks can be estimated at relevant time intervals, 2, 5 and 10 years. Numbers of missing values will be summarised for each factor. Multiple imputation will be used to replace missing values. Discrimination of the final model will be assessed with Harrell's C-statistic. Internal validity will be assessed by bootstrap resampling.

- *Objective 3 – Long term rupture rates*

All patients, including those who underwent aneurysm occlusion, will be included in time-to-event analysis which will cover the whole duration of available follow up. This will include Kaplan-Meier and proportional hazards models for univariate and multivariate survival curve fitting. A cumulative Hazard plot will be used to assess if rupture risk is constant or varies with time from diagnosis.

Once the cohort is established, funding will be sought for repeated searches at regular intervals (5 yearly) to update models and provide progressively longer-term rupture rates.

Patient and public involvement

A workgroup was organised with the Wessex Subarachnoid Haemorrhage Support Group to discuss UIA research where it was confirmed that better decision making on aneurysm treatment is the main concern for patients, but patients do not want to have their management randomised and

For peer review only

therefore an RCT is unlikely to succeed. Consequently, a better understanding of the natural history of UIA was deemed the top priority and that long term, ideally lifetime risks, are what is relevant to patients. This formed the basis of the current study.

During May 2020, while face to face public involvement was not possible due to the COVID pandemic, patients in the neurovascular telephone clinic at University Hospital Southampton were surveyed to assess the study design. All patients strongly supported a study of the natural history of UIA. Although some said they would decline participation in imaging or interventional studies, all confirmed they would be happy for their records to be searched for a natural history study, without full informed consent as is proposed.

Ethics and dissemination

Ethical considerations (including informed consent)

Seeking informed consent from all patients to break confidentiality and transfer their identifiable details is not possible without biasing the results. Patients whose aneurysm ruptures have a high likelihood of death or severe disability which would leave them unable to provide informed consent. If informed consent was mandatory, the final cohort would contain an underrepresentation of patients whose aneurysm ruptured, thus skewing the observed rupture rates.

In order to process patient identifiable data without consent the study has been given conditional support under Section 251 from the HRA Confidentiality Advisory Group (21/CAG/0033). This allows the transfer of patient identifiable data outside of the direct clinical care team for the purpose of this study. The patient identifiable data can thus be transferred to the co-ordinating team who in turn can upload this data to the HES/PEDW/SMD databases. The protocol has also been reviewed by the South Central Hampshire A Research Ethics Committee and issued a favourable opinion in March 2021 (21/SC/0064). The REC and CAG committees will be updated on all significant protocol amendments by the study co-ordinator.

Monitoring

As a study without direct patient contact there will not be a separate data monitoring committee. Instead, this role will be conducted by the trial management committee.

Dissemination and data availability

The results will be disseminated in peer reviewed journals. Authorship will follow International Committee of Medical Journal Editors recommendations and professional writers will not be used. Upon completion of the study, the anonymised dataset will be available both to members of the ROAR collaboration and other external researchers. They will be available from the chief investigator upon reasonable request.

Discussion

For peer review only

Although the natural history of UIA has been previously investigated with multiple prospective cohort studies, the rupture rate has varied significantly between these. The design of the ROAR Study addresses many of the criticisms of these previous natural history studies. ISUIA[7] is the first natural history study and is drawn from a population that is genetically closest to that of the UK. However, its results are subject to selection bias with 71% of their UIA being treated either before inclusion or during follow-up. It is not known if that selection was random or based on a feature associated with risk such as aneurysm irregularity. It is therefore not known how the rupture rates in the remaining potentially lower-risk patients translate to the general population. The UK has a much lower treatment rate of UIA (approximately 20%). This is one of the lowest rates in a developed country making it the ideal setting for a natural history study. It remains however that there will be some treatments performed during the study which inevitably will produce some selection bias that cannot be eliminated. Other countries with lower treatment rates are likely to also have low availability of imaging and hence much lower case identification limiting the cohort size and introducing different biases.

There is also a risk of selection bias in ROAR arising from the methods employed for patient identification. Patients in whom no MDT, clinic notes or radiology report was created would be effectively excluded. These are also the patients more likely to not undergo treatment. However, our survey of UK neurovascular surgeons suggest it is only a very small minority that are not discussed at MDT or seen in a neurosciences clinic. This was also borne out in pilot studies in the development of the protocol. The timeframe for searching patients within a unit's available records creates the potential for introducing prevalence-incidence selection bias. The ROAR Study will mitigate this by identifying patients as *new* or *follow-up* based on whether or not the UIA was diagnosed during that unit's search window and use the identifying document dates accordingly.

The largest UIA studies are based on Japanese populations and the study with the least bias on a Finnish population. Both observed higher rates of aneurysm rupture than ISUIA. Although it has been assumed ethnicity is a risk factor for rupture and therefore included in the PHASES score, it is not known if the different rupture rates in these studies were due to differences in genetics, environment or study design (and consequently biases). The ROAR Study will observe rupture rates in a UK population which removes any concerns over the influence of ethnicity in the results and makes it generalisable for UIA decision making in patients in the UK. Given the use of national level databases ROAR is designed as a single country study and as a result there are limitations to its generalisability outside the UK. However, the UK's population probably more closely resembles most European and North American countries than Japan or Finland.

With the exception of the Juvela study,[9] the follow-up lengths of previous UIA natural history studies are less than 5 years. Patients are generally not interested in such short-term risks and want to know their lifetime risk. Unfortunately, it is not known if risk is constant over time and therefore if these short-term estimates can be extrapolated to a patient's lifetime. The hybrid design of the ROAR Study will immediately generate follow-up periods of up to 15 years per patient. These will still be shorter than the typical 30-year life expectancy of someone diagnosed with a UIA at 50 and there will still therefore be limitations to extrapolation. However, it will be possible to perform repeat

For peer review only

searches of the prospectively maintained databases for hospital admissions and deaths at intervals in the future such that ultimately it will yield realistic lifetime estimates.

Long-term follow up of such a large cohort of patients is only feasible through the use of national databases. A traditional prospective study would be too costly and time consuming to be realistic as well as suffering significant loss to follow up. The risk posed by using national databases is that patients emigrating are not censored when they leave the country. However, these databases record when patients deregister their general practice at which time they will be censored from further analysis.

One of the limitations of using national databases for hospital admissions is that identifying rupture events relies on the accuracy of the hospital coders and the diagnosis codes they assign (both to the primary and secondary diagnoses). The ROAR Study will assess the magnitude of any miscoding by collecting any available follow up data from the neurosciences record. In cases where a confirmed aneurysm rupture occurred, we will examine which hospital codes were assigned to that episode. We will also mitigate this limitation by searching for codes for all types of intra-cranial haemorrhage, not just subarachnoid haemorrhage, and subsequently review the medical records and imaging studies to confirm the true diagnosis for that patient.

Whilst subject to a number of limitations, the ROAR study has mitigations for most of these and will therefore be less susceptible to them than previous studies. It is therefore expected to definitively evaluate the validity of PHASES, assess additional predictors of rupture and assess long term risks of rupture.

Contributors

DB conceived the study. DB, SH, and JB designed the protocol. JB provided statistical support and advised on study design. PG, DD and CH helped piloting and testing the protocol. CT, MT, JP, CU, HP, NG, IA, NT, JvB and JG provided further feedback on the protocol. ROAR Investigators, ROAR Investigators All remaining authors contributed to the set-up of the study, have reviewed the protocol and approved the final manuscript.

Competing interests

None of the authors have any conflicts to declare.

Funding

This study/project is funded by the National Institute for Health Research (NIHR) Research for Patient Benefit programme (NIHR203628). The views expressed are those of the author(s) and not necessarily those of the NIHR or the Department of Health and Social Care.

For peer review only

This work is also supported by: Smile4Wessex (no grant number), and Royal College of Surgeons England (no grant number) and the Polycystic Kidney Disease Charity (PKD-21-03). Neither the sponsor nor any of the funding sources had a role in designing the study protocol. They will also have no role in the analysis of data nor submission of results.

Sponsor contact information

Sponsor: University Hospital Southampton NHS Foundation Trust

Sponsor reference: RHM NEU0390

Contact name: Mrs Sharon Davies-Dear

Contact email: sharon.davies-dear@uhs.nhs.uk

ROAR Collaborators

Frederick Ewbank,[1] Stefan Mitrasinovic,[1] Oliver Croft,[1] Dominic Townsend[1]

Neeraj Kalra,[3] Imogen Kirkpatrick,[3] Nicole Handy[3]

Alex Rossdeutsch,[4] Ellie Courtney,[4] Zoe Stone[4]

James Loan,[5] Anthony Wiggins,[5] Jay Park,[5] Niamh Rafferty[5]

Nathan McSorley,[6] Ajitesh Anand,[6] Armin Nazari[6]

Giannis Sokratous,[7] Basel Taweel,[7] Kartik Goyal[7]

John Norris,[8] Hannah-Marie Culley[8], Mukul Arora[8]

Milo Hollingworth,[9] Megan Burns,[9] Benjamin Armstrong[9]

Harsh Bhatt,[10] David Lowes,[10] Debra Faulkner[10]

Ciaran Hill,[11] Debayan Dasgupta,[11] Shuja Yaqub,[11] Yuzhi Phuah,[11] Olivier Sluijters,[11] Neda Oskooee,[11] Viraj Pamar,[11] Tarek Elmenofi,[11] Justyna Ekert[11]

Alex Fung,[13] Rebecca Legge[13]

Srihari Deepak,[15] Vigneshwar Veerappan,[14] Swarnava Gupta,[14] Sumeet Sasane,[14] May Ting Tan[15]

Lucie Ferguson[16]

Matthew Myers,[4,17] Antonio Bonardi,[17] Holly Tetlow,[17] Charlotte Dunkerley,[17] Malvika Pandey,[17] William Giffin,[17] Iqbal Bin Lokman,[17] Danielle Hurst,[17] Daniel Ahari[17]

Anouk Borg,[18] Thanos Papadias[18]

Kristy Kehoe,[19] Jeremy Cheong,[19] Isabella Davies,[19] Taisha Peplowska[19]

James Ulrich[20] Mohammed Draz,[20] Barbora Krivankova,[20] Katy Homyer,[20] Vivienne Evans,[20] Steven Tominey,[20] Attika Chaudhary,[20] Sabrina Tengku,[20] Eilidh Middleton,[20] Sytske Lub,[20] Hassan Ismahel,[20] Michalina Wilinska,[20] Nicola Duncan,[20] Joanne Igoli,[20] Ritika Sandaram[20]

For peer review only

Rebecca Hodnett,[21] Naadir Nazar,[21] Omar Ouaret Sorr,[21] Fatima Camp,[21] Tom Ferreira[21]

Benjamin Fisher,[22] Devika Rajashekar,[22] Rachel Dumbrell,[22] Ananya Muthukumar,[22] Mohammad Anas,[22] Lilian Nwosu,[22] Ariadne Holmes[22]

Ikenna Ogbu,[23] Madalina Pasca,[23] Veer Patel,[23] Mustafa Dashti,[23] Zeluleko Sibanda,[23] Erin Hwang,[23] Zara Adil[23]

Samir Matloob,[24] Vanessa Chow[24]

Rosa Sun,[26] Aparnu Vimal,[26] Talhah Chaudri,[26] Lauren Baldwin[26]

Haritha Maripi,[27] Balint Borbas,[27] Mariyam Mujeeb[27]

For peer review only

References

1 Vlak, M. H., Algra, A., Brandenburg, R. & Rinkel, G. J. Prevalence of unruptured intracranial aneurysms, with emphasis on sex, age, comorbidity, country, and time period: a systematic review and meta-analysis. *Lancet Neurol* **10**, 626-636, doi:10.1016/S1474-4422(11)70109-0 (2011).

2 Cras, T. Y. *et al.* Determinants of the Presence and Size of Intracranial Aneurysms in the General Population: The Rotterdam Study. *Stroke* **51**, 2103-2110, doi:10.1161/STROKEAHA.120.029296 (2020).

3 de Rooij, N. K., Linn, F. H., van der Plas, J. A., Algra, A. & Rinkel, G. J. Incidence of subarachnoid haemorrhage: a systematic review with emphasis on region, age, gender and time trends. *J Neurol Neurosurg Psychiatry* **78**, 1365-1372, doi:10.1136/jnnp.2007.117655 (2007).

4 Greving, J. P. *et al.* Development of the PHASES score for prediction of risk of rupture of intracranial aneurysms: a pooled analysis of six prospective cohort studies. *Lancet Neurol* **13**, 59-66, doi:10.1016/S1474-4422(13)70263-1 (2014).

5 Nieuwkamp, D. J. *et al.* Changes in case fatality of aneurysmal subarachnoid haemorrhage over time, according to age, sex, and region: a meta-analysis. *Lancet Neurol* **8**, 635-642, doi:10.1016/S1474-4422(09)70126-7 (2009).

6 Algra, A. M. *et al.* Procedural Clinical Complications, Case-Fatality Risks, and Risk Factors in Endovascular and Neurosurgical Treatment of Unruptured Intracranial Aneurysms: A Systematic Review and Meta-analysis. *JAMA Neurol* **76**, 282-293, doi:10.1001/jamaneurol.2018.4165 (2019).

7 Wiebers, D. O. *et al.* Unruptured intracranial aneurysms: natural history, clinical outcome, and risks of surgical and endovascular treatment. *Lancet* **362**, 103-110 (2003).

8 Raymond, J. *et al.* Unruptured Intracranial Aneurysms. A Critical Review of the International Study of Unruptured Intracranial Aneurysms (ISUIA) and of Appropriate Methods to Address the Clinical Problem. *Interv Neuroradiol* **14**, 85-96, doi:10.1177/159101990801400111 (2008).

9 Juvela, S., Poussa, K., Lehto, H. & Porras, M. Natural history of unruptured intracranial aneurysms: a long-term follow-up study. *Stroke* **44**, 2414-2421, doi:10.1161/STROKEAHA.113.001838 (2013).

10 Morita, A. *et al.* The natural course of unruptured cerebral aneurysms in a Japanese cohort. *N Engl J Med* **366**, 2474-2482, doi:10.1056/NEJMoa1113260 (2012).

11 Wermer, M. J. *et al.* Yield of short-term follow-up CT/MR angiography for small aneurysms detected at screening. *Stroke* **37**, 414-418, doi:10.1161/01.STR.0000199077.06390.35 (2006).

12 Sonobe, M., Yamazaki, T., Yonekura, M. & Kikuchi, H. Small unruptured intracranial aneurysm verification study: SUAVe study, Japan. *Stroke* **41**, 1969-1977, doi:10.1161/STROKEAHA.110.585059 (2010).

13 Ishibashi, T. *et al.* Unruptured intracranial aneurysms: incidence of rupture and risk factors. *Stroke* **40**, 313-316, doi:10.1161/STROKEAHA.108.521674 (2009).

14 Delgado-Rodríguez, M. & Llorca, J. Bias. *J Epidemiol Community Health* **58**, 635-641, doi:10.1136/jech.2003.008466 (2004).

15 Hackenberg, K. A. M. *et al.* Definition and Prioritization of Data Elements for Cohort Studies and Clinical Trials on Patients with Unruptured Intracranial Aneurysms: Proposal of a Multidisciplinary Research Group. *Neurocrit Care* **30**, 87-101, doi:10.1007/s12028-019-00729-0 (2019).

16 Woodfield, R., Grant, I., Sudlow, C. L., Group, U. B. S. O. & Group, U. B. F.-U. a. O. W. Accuracy of Electronic Health Record Data for Identifying Stroke Cases in Large-Scale Epidemiological Studies: A Systematic Review from the UK Biobank Stroke Outcomes Group. *PLoS One* **10**, e0140533, doi:10.1371/journal.pone.0140533 (2015).

17 Riley, R. D. *et al.* Calculating the sample size required for developing a clinical prediction model. *BMJ* **368**, m441, doi:10.1136/bmj.m441 (2020).

18 Nurmonen, H. J. *et al.* Polycystic kidney disease among 4,436 intracranial aneurysm patients from a defined population. *Neurology* **89**, 1852-1859, doi:10.1212/WNL.0000000000004597 (2017).

19 Royston, P. Tools for Checking Calibration of a Cox Model in External Validation: Approach Based on Individual Event Probabilities. *The Stata Journal* **14**, 738-755, doi:10.1177/1536867x1401400403 (2014).

For peer review only

SPIRIT

STANDARD PROTOCOL ITEMS: RECOMMENDATIONS FOR INTERVENTIONAL TRIALS

SPIRIT 2013 Checklist: Recommended items to address in a clinical trial protocol and related documents*

Section/item	ItemNo	Description	Reported on page
**Administrative information**			
Title	1	Descriptive title identifying the study design, population, interventions, and, if applicable, trial acronym	1
Trial registration	2a	Trial identifier and registry name. If not yet registered, name of intended registry	3
	2b	All items from the World Health Organization Trial Registration Data Set	3, 12
Protocol version	3	Date and version identifier	3
Funding	4	Sources and types of financial, material, and other support	12
Roles and responsibilities	5a	Names, affiliations, and roles of protocol contributors	12
	5b	Name and contact information for the trial sponsor	12
	5c	Role of study sponsor and funders, if any, in study design; collection, management, analysis, and interpretation of data; writing of the report; and the decision to submit the report for publication, including whether they will have ultimate authority over any of these activities	12
	5d	Composition, roles, and responsibilities of the coordinating centre, steering committee, endpoint adjudication committee, data management team, and other individuals or groups overseeing the trial, if applicable (see Item 21a for data monitoring committee)	N/A
**Introduction**			
Background and rationale	6a	Description of research question and justification for undertaking the trial, including summary of relevant studies (published and unpublished) examining benefits and harms for each intervention	4-5
	6b	Explanation for choice of comparators	N/A
Objectives	7	Specific objectives or hypotheses	5

For peer review only

Trial design	8	Description of trial design including type of trial (eg, parallel group, crossover, factorial, single group), allocation ratio, and framework (eg, superiority, equivalence, noninferiority, exploratory)	5
**Methods: Participants, interventions, and outcomes**			
Study setting	9	Description of study settings (eg, community clinic, academic hospital) and list of countries where data will be collected. Reference to where list of study sites can be obtained	5
Eligibility criteria	10	Inclusion and exclusion criteria for participants. If applicable, eligibility criteria for study centres and individuals who will perform the interventions (eg, surgeons, psychotherapists)	7
Interventions	11a	Interventions for each group with sufficient detail to allow replication, including how and when they will be administered	N/A
	11b	Criteria for discontinuing or modifying allocated interventions for a given trial participant (eg, drug dose change in response to harms, participant request, or improving/worsening disease)	N/A
	11c	Strategies to improve adherence to intervention protocols, and any procedures for monitoring adherence (eg, drug tablet return, laboratory tests)	N/A
	11d	Relevant concomitant care and interventions that are permitted or prohibited during the trial	N/A
Outcomes	12	Primary, secondary, and other outcomes, including the specific measurement variable (eg, systolic blood pressure), analysis metric (eg, change from baseline, final value, time to event), method of aggregation (eg, median, proportion), and time point for each outcome. Explanation of the clinical relevance of chosen efficacy and harm outcomes is strongly recommended	7
Participant timeline	13	Time schedule of enrolment, interventions (including any run-ins and washouts), assessments, and visits for participants. A schematic diagram is highly recommended (see Figure)	N/A
Sample size	14	Estimated number of participants needed to achieve study objectives and how it was determined, including clinical and statistical assumptions supporting any sample size calculations	8
Recruitment	15	Strategies for achieving adequate participant enrolment to reach target sample size	N/A

For peer review only - http://bmjopen.bmj.com/site/about/guidelines.xhtml

**Methods: Assignment of interventions (for controlled trials)**			
Allocation:			
Sequence generation	16a	Method of generating the allocation sequence (eg, computer-generated random numbers), and list of any factors for stratification. To reduce predictability of a random sequence, details of any planned restriction (eg, blocking) should be provided in a separate document that is unavailable to those who enrol participants or assign interventions	N/A
Allocation concealment mechanism	16b	Mechanism of implementing the allocation sequence (eg, central telephone; sequentially numbered, opaque, sealed envelopes), describing any steps to conceal the sequence until interventions are assigned	N/A
Implementation	16c	Who will generate the allocation sequence, who will enrol participants, and who will assign participants to interventions	N/A
Blinding (masking)	17a	Who will be blinded after assignment to interventions (eg, trial participants, care providers, outcome assessors, data analysts), and how	N/A
	17b	If blinded, circumstances under which unblinding is permissible, and procedure for revealing a participant's allocated intervention during the trial	N/A
**Methods: Data collection, management, and analysis**			
Data collection methods	18a	Plans for assessment and collection of outcome, baseline, and other trial data, including any related processes to promote data quality (eg, duplicate measurements, training of assessors) and a description of study instruments (eg, questionnaires, laboratory tests) along with their reliability and validity, if known. Reference to where data collection forms can be found, if not in the protocol	5-7
	18b	Plans to promote participant retention and complete follow-up, including list of any outcome data to be collected for participants who discontinue or deviate from intervention protocols	N/A
Data management	19	Plans for data entry, coding, security, and storage, including any related processes to promote data quality (eg, double data entry; range checks for data values). Reference to where details of data management procedures can be found, if not in the protocol	7
Statistical methods	20a	Statistical methods for analysing primary and secondary outcomes. Reference to where other details of the statistical analysis plan can be found, if not in the protocol	8-9

For peer review only - http://bmjopen.bmj.com/site/about/guidelines.xhtml

	20b	Methods for any additional analyses (eg, subgroup and adjusted analyses)	N/A
	20c	Definition of analysis population relating to protocol non-adherence (eg, as randomised analysis), and any statistical methods to handle missing data (eg, multiple imputation)	8-9
**Methods: Monitoring**			
Data monitoring	21a	Composition of data monitoring committee (DMC); summary of its role and reporting structure; statement of whether it is independent from the sponsor and competing interests; and reference to where further details about its charter can be found, if not in the protocol. Alternatively, an explanation of why a DMC is not needed	10
	21b	Description of any interim analyses and stopping guidelines, including who will have access to these interim results and make the final decision to terminate the trial	N/A
Harms	22	Plans for collecting, assessing, reporting, and managing solicited and spontaneously reported adverse events and other unintended effects of trial interventions or trial conduct	N/A
Auditing	23	Frequency and procedures for auditing trial conduct, if any, and whether the process will be independent from investigators and the sponsor	10
**Ethics and dissemination**			
Research ethics approval	24	Plans for seeking research ethics committee/institutional review board (REC/IRB) approval	9-10
Protocol amendments	25	Plans for communicating important protocol modifications (eg, changes to eligibility criteria, outcomes, analyses) to relevant parties (eg, investigators, REC/IRBs, trial participants, trial registries, journals, regulators)	10
Consent or assent	26a	Who will obtain informed consent or assent from potential trial participants or authorised surrogates, and how (see Item 32)	9-10
	26b	Additional consent provisions for collection and use of participant data and biological specimens in ancillary studies, if applicable	9-10
Confidentiality	27	How personal information about potential and enrolled participants will be collected, shared, and maintained in order to protect confidentiality before, during, and after the trial	7, 9-10
Declaration of interests	28	Financial and other competing interests for principal investigators for the overall trial and each study site	12

For peer review only

For peer review only - http://bmjopen.bmj.com/site/about/guidelines.xhtml

Access to data	29	Statement of who will have access to the final trial dataset, and disclosure of contractual agreements that limit such access for investigators	10
Ancillary and post-trial care	30	Provisions, if any, for ancillary and post-trial care, and for compensation to those who suffer harm from trial participation	N/A
Dissemination policy	31a	Plans for investigators and sponsor to communicate trial results to participants, healthcare professionals, the public, and other relevant groups (eg, via publication, reporting in results databases, or other data sharing arrangements), including any publication restrictions	10
	31b	Authorship eligibility guidelines and any intended use of professional writers	10
	31c	Plans, if any, for granting public access to the full protocol, participant-level dataset, and statistical code	N/A
**Appendices**			
Informed consent materials	32	Model consent form and other related documentation given to participants and authorised surrogates	N/A
Biological specimens	33	Plans for collection, laboratory evaluation, and storage of biological specimens for genetic or molecular analysis in the current trial and for future use in ancillary studies, if applicable	N/A

*It is strongly recommended that this checklist be read in conjunction with the SPIRIT 2013 Explanation & Elaboration for important clarification on the items. Amendments to the protocol should be tracked and dated. The SPIRIT checklist is copyrighted by the SPIRIT Group under the Creative Commons "Attribution-NonCommercial-NoDerivs 3.0 Unported" license.

For peer review only
