# Supplementary material for: Risk of Aneurysm Rupture (ROAR) study: protocol for a long-term, longitudinal, UK multicentre study of unruptured intracranial aneurysms
Source: BMJ Open. 2023 Mar 16;13(3):e070504. doi: 10.1136/bmjopen-2022-070504 (PMC10030903; doi:10.1136/bmjopen-2022-070504)
Supplement: Reviewer comments [file bmjopen-2022-070504.reviewer_comments.pdf]

PEER REVIEW HISTORY

BMJ Open publishes all reviews undertaken for accepted manuscripts. Reviewers are asked to complete a checklist review form (**http://bmjopen.bmj.com/site/about/resources/checklist.pdf**) and are provided with free text boxes to elaborate on their assessment. These free text comments are reproduced below.

ARTICLE DETAILS

**TITLE (PROVISIONAL)**	Risk Of Aneurysm Rupture (ROAR) study: protocol for a long-term, longitudinal, UK multicentre study of unruptured intracranial aneurysms
**AUTHORS**	Hall, Samuel; Birks, Jacqueline; Anderson, Ian; Bacon, Andrew; Brennan, Paul; Bennett, David; Chavredakis, Emmanuel; Critchley, Giles; Dow, Graham; Downer, Jonathan; Galea, James; Grover, Patrick; Gurusinghe, Nihal; Helmy, Adel; Kounin, Gueorgui; Mukerji, Nitin; Patel, Hiren; Patel, Jash; Ross, Nicholas; St George, Jerome; Teo, Mario; Tolias, Christos; Tzerakis, Nikolaos; Uff, Christopher; van Beijum, Janneke; Veighey, Kristin; White, Edward; Whitfield, Peter; ROAR Investigators, Bulters, Diederik; ROAR Investigators

This article was not externally reviewed at BMJ Open. Protocols that have been independently assessed prior to submission to BMJ Open are usually fast-tracked to publication on the grounds that further substantial changes will not be possible. This independent assessment will usually be external, independent review for both a substantial grant award from a non-commercial or government funder and ethics approval.
